# Bioinspired integrated nanosystems based on solid-state nanopores: “*iontronic*” transduction of biological, chemical and physical stimuli

**DOI:** 10.1039/c6sc04255d

**Published:** 2016-10-26

**Authors:** Gonzalo Pérez-Mitta, Alberto G. Albesa, Christina Trautmann, María Eugenia Toimil-Molares, Omar Azzaroni

**Affiliations:** a Instituto de Investigaciones Fisicoquímicas Teóricas y Aplicadas (INIFTA) , Universidad Nacional de La Plata , CONICET , CC. 16 Suc. 4 , 1900 La Plata , Argentina . Email: azzaroni@inifta.unlp.edu.ar; b GSI Helmholtzzentrum für Schwerionenforschung , Darmstadt , Germany; c Technische Universität Darmstadt , Darmstadt , Germany

## Abstract

The ability of living systems to respond to stimuli and process information has encouraged scientists to develop integrated nanosystems displaying similar functions and capabilities. In this regard, biological pores have been a source of inspiration due to their exquisite control over the transport of ions within cells, a feature that ultimately plays a major role in multiple physiological processes, *e.g.* transduction of physical stimuli into nervous signals. Developing abiotic nanopores, which respond to certain chemical, biological or physical inputs producing “iontronic” signals, is now a reality thanks to the combination of “soft” surface science with nanofabrication techniques. The interplay between the functional richness of predesigned molecular components and the remarkable physical characteristics of nanopores plays a critical role in the rational integration of molecular functions into nanopore environments, permitting us to envisage nanopore-based biomimetic integrated nanosystems that respond to a variety of external stimuli such as pH, redox potential, molecule concentration, temperature, or light. Transduction of these stimuli into a predefined “*iontronic*” response can be amplified by exploiting nanoconfinement and physico-chemical effects such as charge distribution, steric constraints, equilibria displacement, or local changes in ionic concentration, to name but a few examples. While in past decades the focus has been mostly on their fundamental aspects and the in-depth study of their interesting transport properties, for several years now nanopore research has started to shift towards specific practical applications. This work is dedicated to bringing together the latest developments in the use of nanopores as “*iontronic*” transducing elements. Our aim is to show the wide potential of abiotic nanopores in sensing and signal transduction and also to promote the potential of this technology among doctoral students, postdocs, and researchers. We believe that even a casual reader of this perspective will not fail to be impressed by the wealth of opportunities that solid-state nanopores can offer to the transduction of biological, physical and chemical stimuli.

## Abstract 2

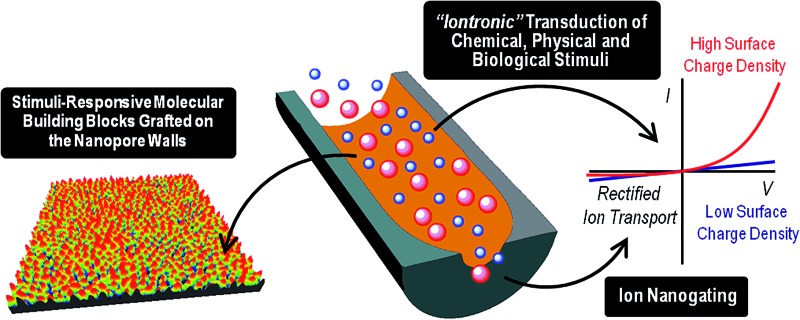
Here, we show the wide potential of abiotic nanopores in sensing and signal transduction and also to promote the potential of this technology among doctoral students, postdocs, and researchers.

## 1. Introduction

Much of the inspiration to construct highly functional architectures comes from the millennial quest of man to look at nature's complete technological design. Biological structures provide a wide range of systems with different functions that ultimately serve as a source of inspiration in materials science. In this way, biomimetic materials research has facilitated numerous avenues to create multifunctional materials by blending concepts from different disciplines.^1,2^ For instance, as we move further into the new century, the convergence of chemistry, physics and nanotechnology seems to indeed offer almost unlimited opportunities for constructing biomimetic nanosystems and devices *via* top-down and bottom-up approaches. In all living systems biological channels work as nanodevices in charge of regulating key functions such as electric potential, ionic flow, and molecular transport across the boundaries of the cells. Furthermore, living organisms utilize signal transduction and signal amplification based on these biological channels for selective and amplified chemical responses towards particular stimuli. From a pragmatic perspective we could say that signal transduction and signal amplification involving the transport of ions through channels comes into play in almost all domains of life. Along these lines, the virtues of working with nanofluidic elements are being increasingly recognized by the scientific community.^3,4^ This has led to the emergence of a research area that is currently at the forefront of materials science and engineering.^5,6^ The advent of new strategies to create single solid-state nanopores has resulted in an increasing mastery of the construction of nanoscale fluidic structures and has given a decisive impetus not only towards the development of this exciting area of nanotechnology but has also opened up new possibilities to reproducibly engineer nanopore and nanochannel architectures with various shapes and diameters down to a few nanometers.^7^ Typical examples are track-etched nanopores,^8^ nanopipettes,^9^ and nanoelectrodes.^10^ This endeavor gave rise to design concepts to construct fully “abiotic” nanochannels with dimensions comparable to those of biological pores.^11^ One major attraction of these nanofluidic elements is their outstanding ability to control and manipulate the transport of chemical and biochemical species flowing through them, thus enabling the construction of ionic circuits capable of sensing, switching, or separating diverse species in aqueous solutions. Furthermore, these nanofluidic devices have also been shown to display transport properties that resemble biological protein ion channels, such as ion selectivity, current rectification, flux inhibition by protons and divalent cations, transport of ions against concentration gradients, and even ion current fluctuations. In the particular case of asymmetric nanochannels/nanopores, appealing rectification effects arise when the channel surface is charged and the dimensions are comparable to the Debye length.^12^ These fascinating physicochemical properties displayed by charged nanochannels or nanopores have provided the impetus to create new functional and addressable nanofluidic architectures and also led to the birth of a whole new area of research concerning the design of nanochannel-based devices relying on surface charge governed ionic transport. This field has been called “*iontronics*”, and it was first mentioned by Han *et al.* ^13^ to describe ion-based information processes and devices^14,15^ that execute their functions in close analogy with electronics – that deals with devices controlling the transport of electrons. However, in order to confer selectivity and manipulate ionic transport through solid-state nanopores, it is necessary to develop and explore new methods for functionalizing the pore walls. Hence, the creation of functional nanopores capable of acting as selective ion channels or smart nanofluidic sensors depends critically on our ability to assemble and build up molecular architectures in a predictable manner within confined geometries with dimensions comparable to the size of the building blocks themselves. To command ion transport functions using nanofluidic devices we need to equip them with additional, artificial gating mechanisms through the integration of specific properties of polymeric, supramolecular and biological materials to benefit from the excluded volume effects, charge distributions and specific functions that these building blocks can confer to the nanopores. In this way we would be able to design nanofluidic systems with the ability to transduce physical, biological or chemical signals into a measurable and controllable ionic current, *i.e.* “*iontronic*” readout.^16^

The use of solid-state nanopores or nanofluidic elements for the development of new paradigms for signal transduction and sensing applications is based on the preparation of systems that are able to respond specifically to a certain target stimulus (physical, chemical or biological) inducing predefined chemical, biochemical or supramolecular changes on the pore walls. The central idea relies on the fact that physical, chemical or biological stimuli could modulate the physicochemical characteristics of the pore wall with a concomitant effect on the ion transport properties.^17^ As such, the synergy arising from the combination of organic, polymeric or even biological components with pre-selected functions and the remarkable physical characteristics of solid-state nanopores can offer new avenues in information processing, sensing, signal transduction and amplification.

Taken all together, the above considerations have strongly motivated the idea to write a review in which the interests of scientists, engineers, postdoctoral fellows and students should be considered. We also wish to present some of the practical technologies that are based on responsive nanofluidic devices to nonspecialists. For the sake of simplicity, the respective nanofluidic systems will be presented according to the signals or stimuli transduced. We stress that despite this arbitrary classification, it is useful to observe the basic physico-chemical principles underlying the behavior of each system and the molecular mechanisms that operate behind the transduction process. We trust that readers will find the basic knowledge and technical results contained herein informative and useful for their work, whether this is for advanced research or for design and construction of such nanodevices. We hope that the present contribution will evidence the multidisciplinary breadth of nanopore research and hence stimulate further advances in this emerging area of nanotechnology.

## 2. Generation of “iontronic” signals – gating mechanisms

A sophisticated reader, casting a glance at the title of this work, might say: “Does such a field of science as “iontronics” exist at all? One could say that the notion of “iontronics” has been around for decades.^18^

A truly iconic predecessor of current nanopore technologies is the Coulter-counting technique^19^ in which small objects passing through a pore trigger a detectable perturbation of the ionic current flowing through it. Later on, during the 1970's, experimental research based on single-channel current recordings came to light as a valuable approach to characterize biological ion channels embedded in lipid bilayer membranes.^20^ This experimental approach permitted the study of biological ion channels that were able to control the passage of ions through the membranes in response to different external stimuli such as specific ions or molecules. One of the most important lessons that we can learn from biological pores is that even though the gating process is mostly mediated by conformational changes, in general, the gating mechanism depends on the stimulus.

In close analogy to biological pores, the presence of specific stimuli can also prompt detectable modulations of the ionic current passing through synthetic nanopores. The readout process based on the measurement of ionic currents is highly affordable and requires very simple instrumentation a compared to other sensing technologies such as surface plasmon resonance, quartz crystal microbalance or fluorescence techniques. In addition, nanopore-based sensing technologies are compatible with the use of nanochannel arrays so that different physical, chemical or biological signals could potentially be processed simultaneously.

**Fig. 1:**
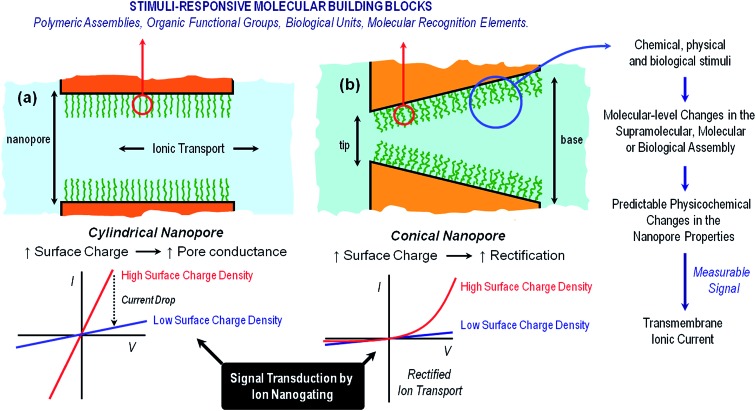
A simplified cartoon describing the configuration of cylindrical (a) and conical (b) nanopores functionalized with stimuli-responsive building blocks. The figure also indicates the expected changes in the transmembrane ionic current upon variations in the environmental conditions leading to high- and low surface charge density states in the responsive layer. Increasing the surface charge leads to larger pore conductance for cylindrical pores (left) and to more pronounced rectification for conical pores (right).

In a typical solid state nanopore setting the readout process involves “steady-state” measurements displaying changes in the current–voltage (*I*–*V*) curves before and after exposing the nanopore to a certain stimulus. In recent years, the “steady-state approach” has gained increasing interest due to its intrinsic experimental simplicity to transduce different specific stimuli into reproducible ionic signals. The primary *I*–*V* response of the nanochannel is highly dependent on its symmetry. The symmetric and asymmetric characteristics of the nanochannels define the symmetric and asymmetric ionic transport properties (Fig. 1).

Symmetric nanochannels, such as cylindrical nanochannels, exhibit symmetric, ohmic-like ionic current properties, *i.e.* linear *I*–*V* curves. On the other hand, asymmetric nanochannels exhibit ionic rectification properties experimentally observed as a non-linear, asymmetric *I*–*V* response. This non-ohmic behavior implies that the magnitude of the ionic currents for applied voltages of one polarity is significantly different from the magnitude of ionic currents for voltages of the opposite polarity.^21^ It is now widely accepted that ionic rectification stems from a broken symmetry in the electric potential that dictates the interaction between the charged pore walls and the ionic carriers, *i.e.* the ions passing through the pore.^22^ This broken symmetry can be observed in charged conical nanopores (geometric asymmetry) but not in charged cylindrical nanopores (geometric symmetry). It has been also demonstrated that inhomogeneous or asymmetric distribution of surface charges on symmetric pores can also confer rectifying properties to the nanofluidic devices.^23^ The sensing and transport properties of asymmetric nanopores are strongly dependent on the shape (or asymmetry) of the pores, *i.e.*, on parameters such as the tip and base diameter or the nanochannel length.^24^ An important feature of charged asymmetric nanopores in comparison to the cylindrical ones is that the voltage drop caused by the nanopore is centered around the narrow tip. In this context, we should mention that, in principle, ion selectivity can only be achieved if the pore diameter is comparable to the thickness of the electrical double layer in a solution of a given ionic strength. Common examples of rectifying nanopores and nanochannels that will be addressed in this work are glass nanopipettes,^25^ track-etched polymer nanochannels,^26^ and silicon nitride nanopores.^27^ The fabrication of suitable channels for “iontronics”, *i.e.* those which are a few nm in size and have controlled geometry, is very challenging. Glass nanopipettes are mostly prepared by standard laser heated pulling techniques, and provide nanochannels with diameters of ∼ 20 nm. Silicon nitride nanopores are prepared by electron beam or focused ion beam sculpting, providing channels of a few nm in diameter and lengths of few tens of a nm. Track-etched polymer single nanochannels are prepared by the unique combination of swift heavy single ion irradiation developed in the 1980s at the GSI Helmholtz Center in Darmstadt (Germany), and subsequent chemical etching, yielding single nanochannels in polymer foils such as *e.g.* polyethylene-teraphthalate (PET), polycarbonate (PC), and polyimide (PI) with excellent control over both pore geometry and size. In addition, the presence of carboxyl groups at the surface of track-etched nanopores enables the application of subsequent chemical modification steps that confer new properties to the nanopores.

In this perspective we refer to work performed mostly on conical and cylindrical track-etched single channels in PET, PC and PI, but examples with glass nanochannels and Si_3_N_4_ pores are also included, and specifically mentioned in the text.

In close resemblance to biological pores, solid state nanopores can also display “gating” properties, *i.e.* opening and closing in response to external stimuli. The use of gated solid-state nanopores for the development of nanofluidic platforms for sensing applications relies on our creativity and ingenuity to create systems that are able to respond specifically to a certain target stimulus that could modulate the ionic transport through the pore. The integration of predesigned building blocks into nanochannels and nanopores can lead to addressable nanofluidic devices. Even though the term “building block” is used to describe a wide variety of functional units, ranging from organic to biological, in our case this term refers to a scenario in which the response of the molecular architectures decorating the nanopore walls can be controlled with accuracy and convenience according to the desired stimulus. This is particularly appealing when we think of a nanochannel as a nanofluidic element with remarkable physical characteristics whose ability to modulate the passage of ions may be precisely adjusted by fine-tuning an external chemical, physical or biological signal. Exciting opportunities are revealed when we think in this manner. Different gating mechanisms provide complementary perspectives from which to consider the manipulation of the ionic transport through the nanopore and ultimately control the generation of iontronic signals in response to external inputs. Harnessing the ability to preconfigure changes in the physicochemical properties of molecular and supramolecular architectures upon exposure to particular stimuli represents the basis of dynamic control over the gating process.^28^ In general, there are three major gating mechanisms that govern the opening and closure of the nanochannels: (a) volume exclusion or steric effects, (b) modulation of surface charge, and (c) wettability switching.

**Fig. 2:**
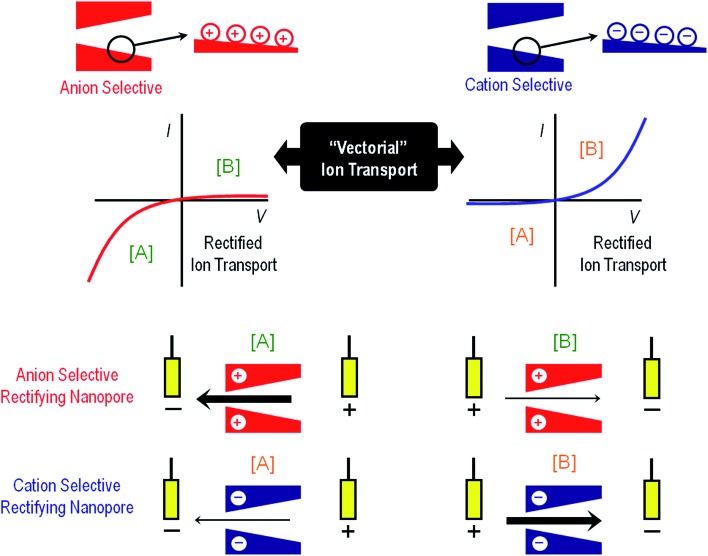
An illustration to indicate the preferential ion flux through positively and negatively charged conical nanopores under different polarization conditions ([A] and [B]). The arrows indicate the direction of the electric current under the applied potential and, therefore, always point in the direction of the movement of positive ions. Thick (thin) arrows indicate the direction of the current in the high (low) conductance state. The current–voltage curves of the nanopores are dictated mainly by the properties of the pore tip, which is the part of the nanopore with higher impedance. If the pore is positively charged, the high conductance state is obtained when the majority carriers (anions) first enter the nanopore tip. This corresponds to *V* < 0 according to the sign criteria. On the other hand, if the pore is negatively charged, the high conductance state is found again when the majority ions (cations) first enter the nanopore tip. This occurs now for *V* > 0. Explanations of the mechanisms of this behavior can be found elsewhere.^29^

*Volume exclusion* effects can stem from conformational transitions or reorganization of molecular units grafted on the pore walls. The binding of analytes to the pore walls can also cause significant volume exclusion effects when the bound molecules are large enough compared to the pore opening to cause a partial or even complete occlusion of the pore. In this case, the current change is proportional to the decrease of the effective nanochannel cross-section. *Modulation of surface charge* represents another gating mechanism commonly used to transduce external stimuli into ionic signals. Electrostatics-based effects may be more versatile compared to the steric approach, especially in the case of asymmetric nanochannels, because the magnitude and the nature of the surface charges dressing the nanopore walls can determine not only the magnitude of the ionic current but also the direction of the rectified current, *i.e.* “vectorial” ion transport (Fig. 2). Depending on the geometric characteristics of the asymmetric nanochannel and the surface density of charged groups on the channel walls, these nanofluidic elements behave as ionic diodes displaying very high rectification efficiencies, *f*_rec_, this parameter corresponding to the ratio of currents recorded for voltages of one polarity and currents recorded for voltages of the opposite polarity. Due to the fact that the ionic current passing through these diodes strongly depends on the surface charge density, subtle changes of the surface charge due to the influence of an external stimulus, *e.g.* analyte binding at the pore tip, should be immediately translated into a change of the rectification efficiency *f*_rec_. In the case of symmetric nanochannels it should be considered that surface charges induce electrostatic screening and electrokinetic effects that may have large effects on the channel conductance. As a result, the modulation of surface charges in symmetric nanochannels due to the influence of an external signal should be detected as a change in the slope of ohmic-like *I*–*V* plots.

Regarding the *wettability switching* mechanism, we should bear in mind that a large number of biophysical studies have demonstrated that the hydrophobicity of a pore can result in a highly effective barrier to ion permeation.^30^ The unfavorable interactions between the hydrophobic pore walls and water molecules in the solution may lead to “dewetting” of the nanochannel, thus leading to an energetic barrier to ion conduction. This process, also known as “hydrophobic gating”, is exploited by biological ion channels to regulate ion flow within their pores. Similarly, in the case of synthetic nanochannels or nanofluidic devices, it has been demonstrated that significant changes in ionic conductance, ON/OFF switching, can be detected when the pore surface is switched from a dewetted, hydrophobic state to a hydrophilic state in which nanochannel is filled with solution.^31^ Hence, the modulation and switching of wetting properties in response to a particular stimulus can be exploited as an interesting gating mechanism to control ionic currents flowing through the nanochannel.

## 3. Environmental signals that trigger nanogating processes in nanopore-based fluidic devices

### Ion-responsive solid-state nanopores

One of the first attempts to modulate the transport properties of nanochannels exploiting specific interactions of ions was reported by Siwy and co-workers.^32^ These authors developed a calcium-induced voltage gating nanochannel by the addition of small amounts of divalent cations to a buffered monovalent ionic solution, which results in an oscillating ionic current through a conical nanochannel.^33^ This behaviour is caused by the transient formation and redissolution of nanoprecipitates, which temporarily block the ionic current through the pore.^34^ Meanwhile, single conically shaped nanopores produce stable ion current fluctuations when in contact with weakly soluble calcium hydrogen phosphate (CaHPO_4_) in solution. In this case, negative surface charges on the walls of conical nanopores in combination with an externally applied voltage lead to a local increase in the ionic concentration in the pore, resulting in precipitation of the salt. As a consequence, the pore spontaneously switches between high and low conductance states, or “open” and “closed” states, respectively.^35^ Similar results were also obtained in the presence of Mg^2+^ and Co^2+^ due to the formation of hydrogen phosphate-based precipitates. The same group demonstrated that, in the presence of soluble salts, multivalent cations like Ca^2+^ or trivalent cobalt sepulchrate can produce localized charge inversion and change the effective pore surface charge profile from negative to positive with a concomitant effect on the rectification properties.^36^ Ion-induced nanoprecipitation in nanofluidic devices was also studied by Pourmand and co-workers using quartz nanopipettes demonstrating that these systems have the ability to control and measure the kinetics of salt precipitation at the nanoscale.^37^

Later, Jiang and co-workers developed an interesting approach to create K^+^-responsive nanochannels based on the use of G quadruplex (G4) DNA.^38^ The operation’s principle relied on the potassium-responsive conformational change of G quadruplex DNA anchored on the nanochannel walls that modulate the effective cross-section (and conductance) of the nanofluidic element. The specific response to K^+^ was demonstrated in control experiments using Li^+^ ions. Experiments performed on bare nanochannels revealed that conductance values were similar regardless of using K^+^ or Li^+^. However, there were remarkable differences when similar experiments were performed on the G4 DNA-modified nanochannels. This biomimetic nanochannel displays a nonlinear response to potassium ions over a concentration range of 0 to 1500 μM.

**Fig. 3:**
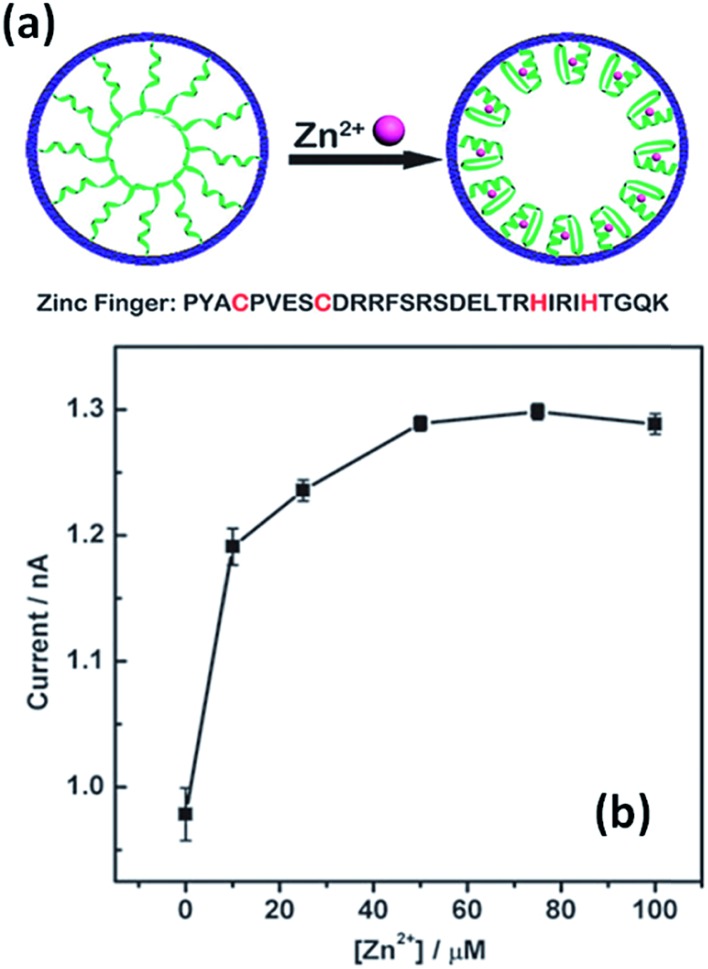
An illustration depicting the conformational changes undergone by the zinc fingers immobilized on the nanochannel upon exposure to Zn^2+^ ions. Current *versus* concentration plot corresponding to an asymmetric nanopore derivatized with zinc fingers in the presence of increasing amounts of Zn^2+^. Reproduced with permission from Tian *et al.*, *Chem. Commun.*, 2010, **46**, 1682–1684. Copyright 2010 Royal Society of Chemistry.

The use of specific interactions between biological entities and metal ions represents an attractive route to develop ion-responsive nanochannels. For instance, Jiang's group extended this concept to the detection of different ionic species. A biomimetic zinc activated ion channel was prepared by incorporating a zinc responsive peptide (“zinc fingers”) into a single polymeric nanochannel.^39^ Zinc finger peptides can undergo significant conformational changes in the presence or in the absence of zinc. In the absence of zinc, few ions pass through the channel which is transduced as a low current state. However, upon zinc binding, zinc fingers fold to finger-like conformations, yielding an increase in the effective channel diameter (Fig. 3). The activation of the ion channel is observed as an increase in transmembrane ionic current. A rather similar concept was employed to detect Hg^2+^ using single-stranded DNA (ssDNA) with an ion-specific thymine–thymine (T–T) base to form strong and stable T–Hg^2+^–T complexes.^40^

Nanochannels modified with 50-thiol-ended T-rich ssDNA exhibit a low current state because the voltage-driven transport of electrolytes across the channel was blocked by the stretched T-rich ssDNA. When the derivatized nanochannel was exposed to Hg^2+^ ions, the current increased due to an increase in the effective cross section of the pore as a result of conformational change generated by the T–Hg^2+^–T complexes, *i.e.* a Hg^2+^ induced conformational change. This gating mechanism was very sensitive and specific to Hg^2+^ with a detection limit of 8 nM. Applying a similar approach, polymeric nanochannels modified with C-rich ssDNA^41^ and DNAzymes^42^ were used to build-up Ag^+^- and Pb^2+^-responsive nanofluidic elements, respectively.

**Fig. 4:**
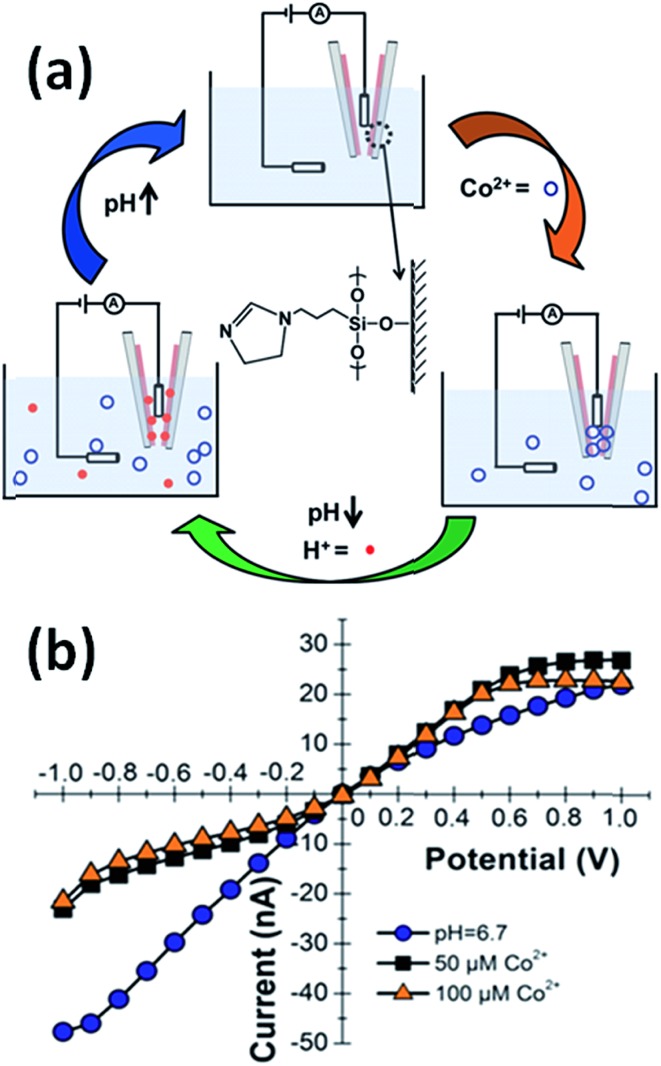
(a) Schematic representation of the Co(ii)-responsive dihydroimidazole (DHI)-modified nanopipette and the corresponding regeneration process in acidic conditions. (b) Current–voltage response of a DHI-modified nanopipette in the absence of Co^2+^ ions (blue dots) and in the presence of 50 μM (black squares) and 100 μM (orange triangles) Co(ii) ions. Measurements were performed in 0.1 M KCl, 0.1 M phosphate buffer (pH = 6.7). Reproduced with permission from Sa *et al.*, *Anal. Chem.*, 2010, **82**, 9963–9966. Copyright 2010 American Chemical Society.

Baker's group demonstrated that quartz nanopipettes modified with imidazole-terminated silane monolayers respond to Co^2+^ in solution.^43^ The response of dihydroimidazole (DHI)-modified nanopipettes was evaluated through examination of the ion current rectification ratio revealing that the binding of Co^2+^ ions induced detectable changes in the current–voltage response. Binding of metal ions to imidazole was reversible through changes in pH in order to regenerate the sensing properties of the device (Fig. 4).

The immobilization of the prion peptide (PrP) on quartz conical nanopipettes was explored as a strategy to create Cu^2+^ sensing platforms. PrP is a copper binding protein with an ability to coordinate several Cu^2+^ equivalents in their N-terminal segments.^44^ The prion peptide was immobilized by simple physisorption onto the negatively charged nanopipette walls without affecting its intrinsic recognition properties. Experiments revealed that the ionic current dropped as Cu^2+^ ions in the micromolar range were chelated by histidine moieties of the peptides whereas blank experiments performed on bare nanopipettes showed no response to Cu^2+^ in up to mM concentrations. A similar strategy was employed to detect Ca^2+^ ions using calmodulin-modified nanopipettes.^45^ Binding of Ca^2+^ to calmodulin was rapidly reversible in neutral buffer requiring no change in conditions to regenerate the receptor.

The integration of supramolecular architectures into nanofluidic elements has gained a great amount of attention in recent years for its powerful contribution to the creation of ion-responsive platforms. For example, biomimetic Zn^2+^-responsive nanochannels were constructed by covalently immobilizing a metal-chelating ligand, 2,2′-dipicolylamine (DPA), in their inner pore walls. The DPA-modified nanochannels showed specific recognition of Zn^2+^ ions compared to the response of the same device in the presence of other metal ions. The grafted DPA molecules on the pore walls act as specific-receptor binding sites for Zn^2+^ ions, which lead to the highly selective Zn^2+^-response as evaluated from the ionic current response.^46^ The formation of Zn^2+^–DPA complexes prompts a significant decrease in the transmembrane ionic current. Furthermore, additional experimental evidence demonstrated that Zn^2+^ chelated track-etched nanochannels can be also used as secondary sensors for HPO_4_^2–^ anions.

In a similar vein, the immobilization of the fluoride-responsive functional molecule, 4-aminophenyl-boronic acid, on a single conical polymer nanochannel led to the development of a fluoride-driven ionic gate.^47^ Experiments confirmed that when the modified nanopore was in the presence of F^–^, negatively charged monofluoride adducts (RB(OH)_2_F^–^), difluoride adducts (RB(OH)F_2_^–^), and trifluoride adducts (RBF_3_^–^) were formed on the pore walls depending on the fluoride concentration. The formation of these species triggered significant changes in the wettability and surface charge of the pore walls. As a consequence, the nanochannel can switch between “ON” and “OFF” states depending on the fluoride concentration. The generation of fluoride-responsive nanofluidic devices was also studied through the incorporation of 1,3-dipropargylaza-*p-tert*-butyl calix[4]crown (C4CE) into polymeric nanochannels.^48^ These nanosystems display high selectivity towards F^–^ sensing in aqueous solution and the sensing platform can be recycled by removing F^–^ with Ca^2+^ ions.

More recently, Jiang and co-workers designed a nanofluidic diode that exhibits tunable gating and rectifying properties in the presence of carbonate ions. This ion-responsive nanodevice was developed through the modification of polymeric nanochannels with 1-(4-amino-phenyl)-2,2,2-trifluoro-ethanone (APTE).^49^ The ionic current passing through the device can be controlled with different concentrations of carbonate ions in solution. The switching of the ionic currents was attributed to the reversible reactions between the hydrophobic APTE molecules and carbonate ions, which control the states of the nanofluidic diode by interplay between charge confinement and dewetting/wetting processes taking place in the pore environment. Interestingly, the APTE-modified nanochannel can be recycled by removing carbonate ions with ionophore CI VII. Due to the high selectivity and sensitivity towards carbonate ions, this nanofluidic diode may find applications in carbon dioxide detection.

In 2015, Jiang's and Azzaroni's groups proposed an interesting twist to the creation of ion-responsive nanofluidic devices by integrating crown ethers into polymer nanochannels. Crown compounds are prominent elements in supramolecular host–guest chemistry as they can bind alkali and alkaline earth cations in a very specific manner in aqueous environments.^50^ The attractive features of host–guest chemistry in combination with nanofluidic devices relies on the fact that the concentration of a pre-selected cation can be responsible for setting well-defined electrostatic conditions on the pore walls. For example, the use of specific host–guest interactions between 18-crown-6 units and potassium ions^51^ (or 4′-aminobenzo-15-crown-5 and sodium ions^52^) can control the charge density on the pore wall with a concomitant influence on their transport and rectification properties.

**Fig. 5:**
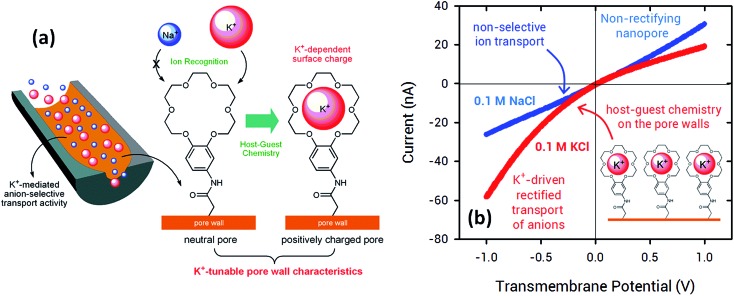
(a) Schematic illustration corresponding to the asymmetric nanochannel modified with 18-crown-6 units. (b) *I*–*V* plots of an asymmetric nanochannel derivatized with 18-crown-6 units in the presence of 0.1 M NaCl (blue trace) and 0.1 M KCl solutions (red trace). Reproduced with permission from Pérez-Mitta *et al.*, *Nanoscale*, 2015, **7**, 15594–15598. Copyright 2015 Royal Society of Chemistry.

Fig. 5 shows *I*–*V* curves of a single asymmetric nanochannel modified with 18-crown-6 units in the presence of NaCl and KCl solutions. The direction and the magnitude of the rectified ionic current in asymmetric channels is dependent on the polarity and magnitude of surface charges. Nanochannels fully derivatized with crown ethers bear no effective negative charges on the pore walls. Then, considering that Na^+^ ions are not complexed by 18-crown-6 groups, polarization of the nanochannel in the presence of 0.1 NaCl reveals no rectification effects (Fig. 5). Conversely, when NaCl is replaced by KCl in the electrolyte solution it can be observed that the channel surface charge was switched from neutral to positive, resulting in the rectified passage of anions through the nanopore, *i.e.* anion selectivity. In other words, the host–guest ion recognition process taking place on the nanopore walls is responsible for the generation of a tunable nanofluidic device with K^+^ dependent rectifying properties.

In this way, the use of crown ethers as recognition elements opens the door for the facile design of ionic gates. These nanoarchitectures can switch from “ON” to “OFF” states or “rectifying” to “nonrectifying” states in the presence of specific chemical stimuli to control ion conduction through the nanochannel. In particular, Na^+^ and K^+^ activated ionic gates might find interesting applications in biosensing and drug delivery based on the relevance of these ionic species in biological systems.

### Thermally activated nanochannels

**Fig. 6:**
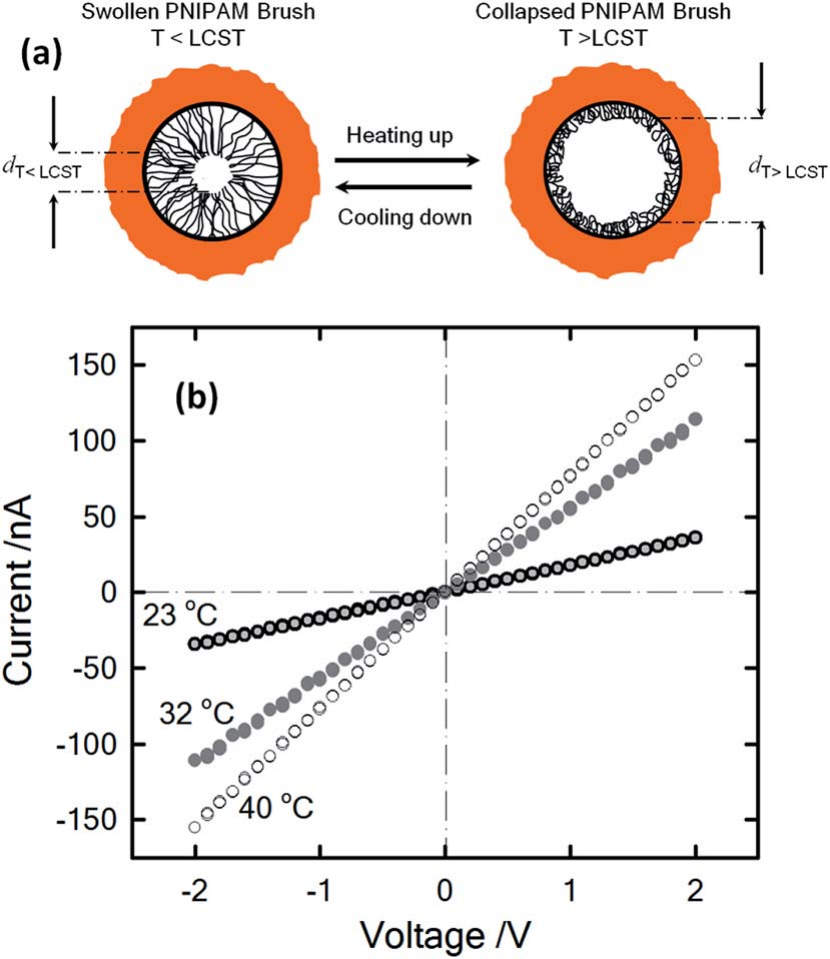
(a) Illustration describing the thermally driven nanoactuation of the polyNIPAM brushes in the PI nanopore. (b) *I*–*V* plots of polyNIPAM-modified nanopores measured in 1 M KCl at different temperatures. Reproduced with permission from Yameen *et al.*, *Small*, 2009, **5**, 1287–1291. Copyright 2009 Wiley-VCH Verlag GmbH & Co. KGaA.

One stimulus of particular interest in biological and non-biological systems is temperature. Biological ionic channels activated by temperature changes transduce this information into conformational changes that open the channel pore. With the inspiration of examples from nature, different research groups built-up biomimetic nanopores displaying thermoactivated ionic transport using macromolecular architectures as functional building blocks. Pioneering experiments by Reber *et al.* ^53^ involved the integration of thermoresponsive poly-*N*-isopropylacrylamide hydrogels into track-etched nanopores to control molecular transport through multipore membranes. These studies sparked the interest of different groups into the development of thermoresponsive membranes.^54^ Later on, this concept was further extended to the creation of thermoresponsive nanofluidic devices. In 2009, Yameen *et al.* ^55^ described the modification of single PI track etched nanopores with poly(*N*-isopropylacrylamide) (PNIPAM) brushes in order to create a thermo-responsive nanochannel. These authors demonstrated that the PNIPAM-modified nanopores exhibit a well-defined and fully reversible thermoactive gating with closure stages that can be controlled by simply tuning the working temperature in the 23–40 °C range (Fig. 6). A similar approach was reported by Jiang and co-workers^56^ employing rectifying conical nanopores modified with PNIPAM brushes. They observed that asymmetric PNIPAM-modified nanochannels can be switched between an ionic rectifying state below 34 °C and the non-rectifying state above 38 °C. The molecular origin of this switching effect was attributed to the conformational collapse of the PNIPAM chains that increases the polymer chain density on the gold surface, resulting in either a reduction in the number of adsorbed anions or an effective screening of the surface charges. Achieving accurate control of the gating properties of nanofluidic devices through subtle temperature changes is an interesting feature that might find applications in “intelligent” devices enabling the temperature-tuned release of drugs.

### Light-responsive nanopores

**Fig. 7:**
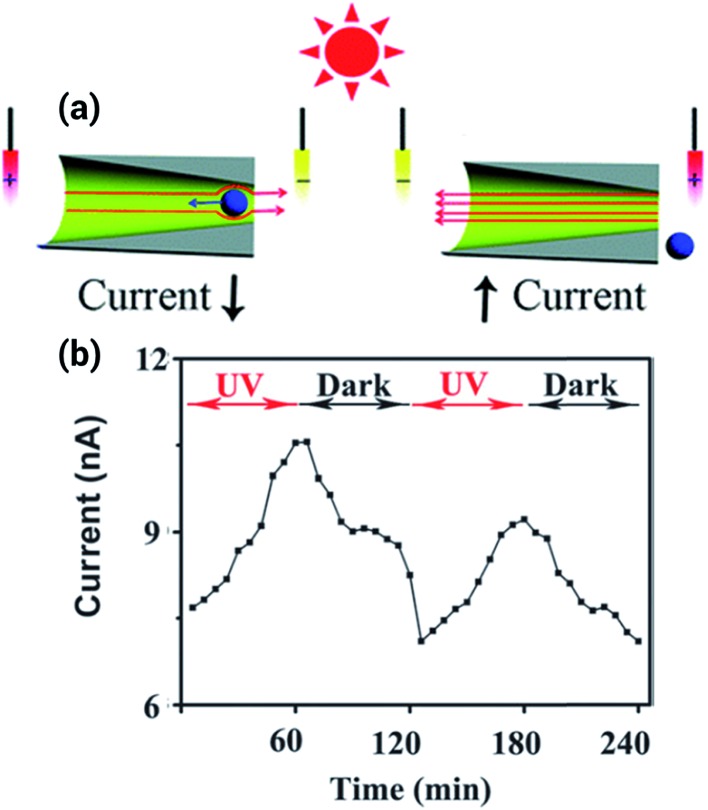
(a) Scheme depicting the working principle of the light responsive nanopore. In presence of ultraviolet light the photoacid deprotonates, increasing the ionic current. (b) Ionic current increase and decrease upon exposure to UV light. Reproduced with permission from Zhang *et al.*, *Chem. Commun.*, 2013, **49**, 2284–2286. Copyright 2013 Royal Society of Chemistry.

Light-responsive nanofluidic devices offer the interesting possibility of gating the ionic current by turning on/off a light source. One of the first experiments concerning this topic was reported by Zhang *et al.* who used titanium dioxide nanotubes that change their charge state in the presence of ultraviolet light to contribute towards the regulation of the transport within the pores.^57^ By using a different approach, the same group showed a system where the regulation of the transport stemmed from the solution instead of from the surface of the pore. To achieve this goal, a photoacid, 8-hydroxypyrene-1,3,6-trisulfonate, was used as an electrolyte. This photoacid deprotonates in the presence of ultraviolet light, producing an increase in the ionic current that was even higher than for the titanium dioxide nanopores (Fig. 7).^58^

However, the lack of examples in the literature regarding systems with a pronounced gating response to light proves that the design of such systems is challenging. In this regard, one approach that provided a remarkable light responsiveness was the modification of the surface of track-etched polyimide nanopores with a malachite-green derivative, which provided not only light but also pH sensitivity.^59^ The principle of operation for this nanofluidic system relies on the fact that UV irradiation or acidification can change the pore walls' properties from neutral (nonselective) to positively charged (anion-selective) by releasing hydroxide ions. This change, in turn, triggers conductance, switching from an OFF state to an ON state. When UV irradiation was interrupted or the solution is alkalinized, the channel became non-selective, and the OFF state was restored. Even after several cycles the photoactive nanochannels exhibited no decay in the ionic gating behavior, showing good reproducibility and reversibility.

Using a similar approach, a system with an even better responsiveness was developed by modifying a nanopore with the spiropyran, 1′-(3-carboxypropyl)-3′,3′-dimethyl-6-nitro-spiro[2*H*-1]benzopyran-2,2′-indoline (SP). In this case, light triggers the opening of the SP molecule, changing its state from a hydrophobic molecule to an ionizable molecule. This combined change in the hydrophobicity and charge of the molecule stimulates the gating process from a closed state in darkness to an open state in the presence of light. Since the SP molecule can have both a positive and a negative charge depending on the pH, it is also a pH sensitive nanodevice.^60^ At pH 7, the nanochannel is in the closed “OFF” state when UV light is off. Upon UV irradiation the nanochannel walls become anionic and consequently, due to ion exclusion, the ionic current is transported by cations. On the other hand, under acidic conditions, the nanochannel is in the closed “OFF” state when UV light is off; but upon UV light irradiation the photoinduced transformation of the SP groups leads to positive charges on the nanochannel walls and anions become the majority carriers.

### Pressure-driven nanogating processes in nanopores

**Fig. 8:**
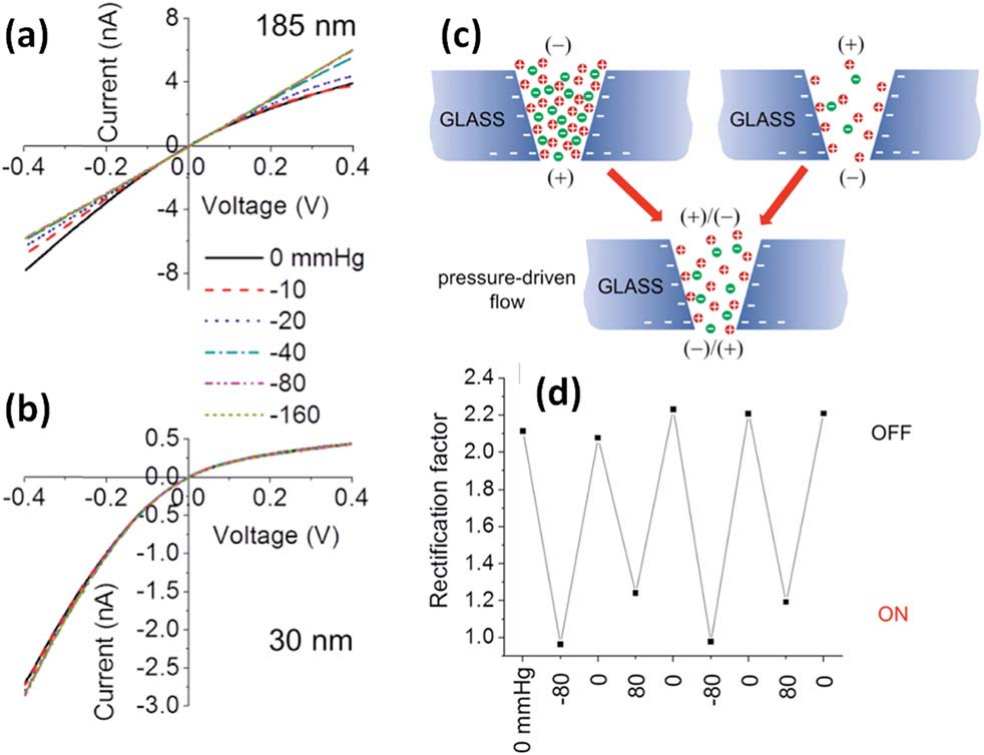
Pressure-dependent *I*–*V* responses of conical-shaped glass nanopores with radii of (a) 185 and (b) 30 nm measured in a 0.01 M KCl solution containing 0.1 mM PBS buffer (pH 7.3). (c) Schematic illustration of the ion distribution close to the tip of a negatively charged conical glass nanopore under different polarization conditions and in the absence and in the presence of pressure-driven flow. (d) Evolution of the rectification factor during pressure switching. Reproduced with permission from Lan *et al.*, *J. Am. Chem. Soc.*, 2011, **133**, 13300–13303. Copyright 2011 American Chemical Society.

It is well known that mechanical stresses modulate a variety of physiological functions, so physical strain can activate mechanosensitive ion channels at the cell surface as well.^61,62^ Within this context, it is interesting to develop nanofluidic devices capable of transducing mechanical forces into ionic signals. In 2011, White and collaborators demonstrated that the ionic rectification in conical-shaped glass nanopores could be modulated using pressure as an input parameter.^63^ The rectified ion transport passing through conical-shaped glass nanopores in low ionic strength solutions was shown to be dependent on the rate of pressure-driven electrolyte flow through the nanopore. This interesting phenomenon was highly dependent on the radius of the nanopore tip and was attributed to the pressure-induced disruption of cation and anion distributions at equilibrium within the nanopore. Fig. 8 shows the *I*–*V* plots for nanopores with different tip radii of 185 and 30 nm. In both cases, in the absence of pressure, the nanopores displayed a marked nonlinear response. This rectifying behavior stems from the presence of residual negative charges on the glass surface of the conical nanopore. It can be seen that when pressure is applied across the larger pore (185 nm) the system exhibits an ohmic *I*–*V* response, *i.e.* the rectification vanishes. The switching can be reversed upon varying the applied pressure (Fig. 8). On the contrary, when a similar experiment was performed on the smaller pore (30 nm) no changes in the rectification properties were observed. These results were rationalized considering that the flow rate is proportional to the third power of the nanopore tip radius, and consequently the pressure-driven flow can only exert considerable influence on large pores of ∼200 nm. These experiments not only introduce new insights into the relationship between fluid flow and ion fluxes in an electrically charged nanopore but also offer interesting avenues to design pressure-responsive nanofluidic devices.

### Electrically addressable nanofluidic devices

An interesting stimulus to control the transport in nanofluidic devices is the electric potential, especially when applied directly to the membrane that contains the nanopores. Martin and Siwy *et al.* reported a PC nanochannel that can rectify the ion current *via* an electrophoretic “insertion and removal” mechanism of single-stranded DNA molecules attached to the nanochannel walls.^64^ According to these authors the rectification and switching mechanism in these nanochannels originates from the voltage-induced electrophoretic insertion of the DNA chains into (OFF-state) and out of (ON-state) the nanopore tip. The OFF-state is obtained because when electrophoretically inserted into the pore tip, the oligonucleotide chains partially occlude the pathway for ion transport, resulting in a low conductance state. Then, by reversing the polarity the chains are electrophoretically removed from the pore tip leading to an “ON” state.

**Fig. 9:**
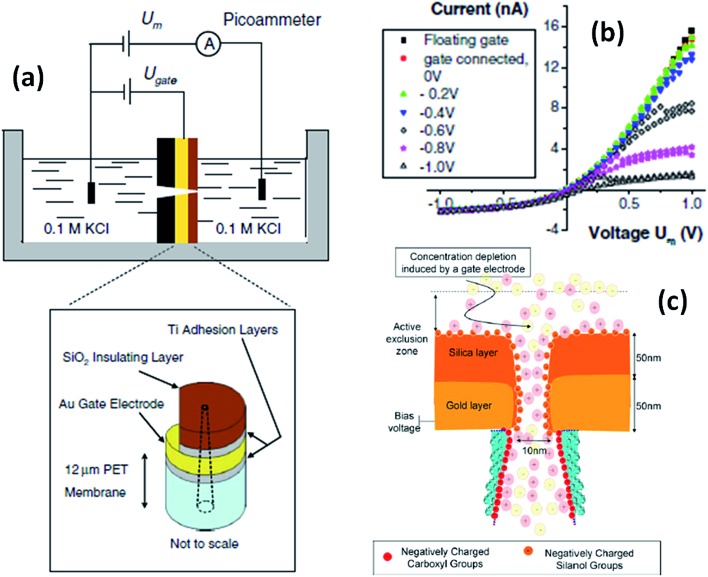
(a) Schematic depiction of the experimental set-up used for studying single conical nanopores containing a Au gate electrode. (b) Current–voltage plots of a single conical nanopore (tip size = 10 nm) with a gate placed at the narrow opening of the pores. The parameter *U*_m_ refers to the voltage applied across the membrane. The legend indicates the gate voltages, *U*_gate_. (c) Simplified cartoon describing the concentration polarization induced by the metal gate present at the small opening of a conical nanopore. Reproduced with permission from Kalman *et al.*, *Anal. Bioanal. Chem.*, 2009, **394**, 413–419. Copyright 2009 Springer-Verlag.

On the other hand, if the pores are coated with a conductive or voltage responsive layer, then fine tuning over the transport behavior of the pores can be achieved. One of the appealing aspects of this particular stimulus is that the voltage can be easily applied and controlled using rather simple setups and therefore is highly useful for designing responsive devices. One of the first experiments performed in this direction was reported by Kalman *et al.* ^65^ where an electrically addressable isolated gold gate was located in the small opening of a conical track-etched nanopore through an electron beam evaporation process. The complete modification procedure consisted of successive layers of titanium, gold, titanium and then silicon dioxide (Fig. 9). After modification, different gate voltages were applied while recording the current–voltage characteristics of the pores. For negative applied voltages, a gating effect was observed for positive transmembrane voltages. In particular, by changing the electric potential applied to the “gate,” the ionic current passing through the pore was switched from a rectifying to a nonrectifying regime. This current modulation is obtained with gate voltages lower than 1 V. The mechanism for this potential-induced modulation of ionic transport was attributed to the enhancement of concentration polarization induced by the gate. Or, in other words, the local electric field generated by the gate affects the ionic distributions at the pore entrance so that concentration polarization takes place.

Another approach to develop electrically addressable nanofluidic devices involves the use of electrochemistry as a key enabling tool to manipulate the generation of surface charges on the nanochannel walls. The first attempts to control the passage of ions through nanopores using redox tunable groups were reported by Martin and his coworkers.^66^ These authors convincingly demonstrated that the decoration of nanochannels with redox-active ferrocene moieties permits the electro-modulated selective transport of cations through the nanofluidic device.

**Fig. 10:**
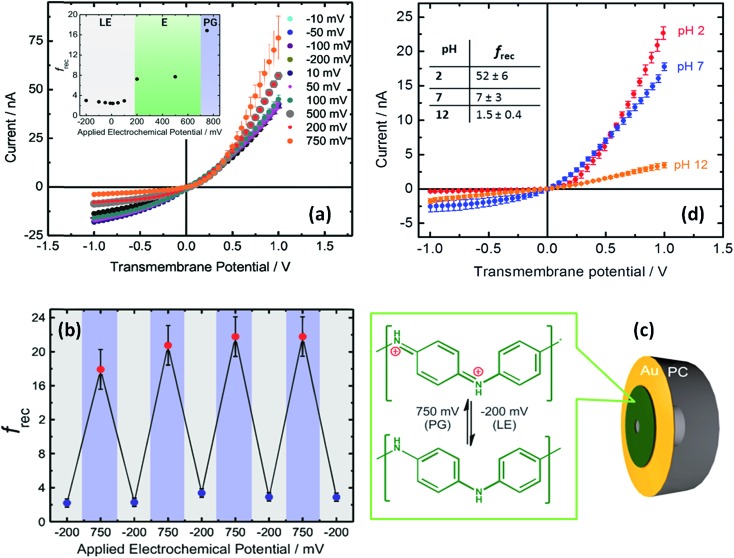
(a) *I*–*V* plots corresponding to a PANI-modified asymmetric nanochannel after different applied potentials at pH 7. The inset describes the changes in the rectification factor upon varying the redox state of PANI: leucoemeraldine (LE), emeraldine (E), and pernigraniline (PG). (b) Reversibility test for PANI-modified asymmetric nanochannels measured at pH 7. (c) Schematic representation of the metallized nanopore used for the electrochemical polymerization of aniline. The chemical changes taking place in the polymer layer during redox cycling are also indicated. (d) *I*–*V* plots corresponding to a PANI-modified asymmetric nanochannel measured under different pH conditions. Reproduced with permission from Pérez-Mitta *et al.*, *J. Am. Chem. Soc.*, 2015, **137**, 15382–15385. Copyright 2015 American Chemical Society.

The use of built-in electrochemically addressable surface charges to gain control over the rectification properties of asymmetric nanochannels was further extended to the integration of conducting polymers into asymmetric nanochannels. Recently, Pérez-Mitta *et al.* ^67^ developed an electrochemically-addressable nanopore device through electropolymerization of aniline onto a metallized polycarbonate conical nanopore (Fig. 10). In this approach, the electrochemically-driven redox conversion of the conducting polymer layer defines the magnitude of the surface charges on the pore walls. Since the p*K*_a_ of polyaniline (PANI) is different for each oxidation state, for a fixed pH the conductance and rectification of ionic current can be tuned with an externally applied voltage. In this case, three different switching states were found which correspond to the chemical states of polyaniline, namely leucoemeraldine, emeraldine and pernigraniline.^68^ Furthermore, due to the fact that the acid–base equilibrium of PANI plays a role in the generation of surface charges, this nanofluidic device is also pH responsive. As a result, the magnitude of the electrochemically generated surface charges arising from the coupling of the proton binding and the applied electrochemical potential is responsible for controlling the ionic current and the rectification properties of the nanofluidic diode. Considering the high degree of control that conducting polymers exert over the switching and gating properties of the devices, it is expected that the integration of redox active polymers into asymmetric nanopores can lead to new designs of switchable nanofluidic diodes with potential applications in biosensing, and energy conversion, among other areas.

### pH-Responsive nanofluidic devices

The use of the local proton concentration as a strategy to manipulate the transport properties of nanofluidic devices has been explored by several groups over the years. One of the first examples was reported by Siwy *et al.* using single conical nanochannels.^69^ They observed that nanochannels exhibiting pH-dependent ionization of the polymeric pore walls led to the rectification of the ionic current passing through the pore. For example, conical PET nanochannels can show pH-modulated rectifying properties because during the etching process carboxyl groups are generated on the inner surface.^70–72^ When the pH of the electrolyte was higher than the p*K*_a_ of the carboxyl groups exposed on the etched polymer surface, the pore surface became negative due to the ionization of the surface groups. The ionization process turned the nanopore into a cation-selective device in which the rectified transport could almost exclusively be ascribed to the passage of cations through the pore tip. However, when the pH was decreased below the p*K*_a_ of the carboxyl groups, the protonation process led to neutralization of the pore with concomitant disappearance of the rectification properties. It is worth mentioning that these pH-responsive properties of “bare” nanochannels are not circumscribed solely to the nanochannels created on PET foils — other materials such as polyimide (PI) nanochannels^73^ or even glass nanopipettes^74^ exhibit similar properties. Since then, many groups have exploited the integration of pH-dependent moieties into nanofluidic devices as a route to manipulate their transport properties. Karhanek and co-workers^75^ described the modification of quartz nanopipettes with poly-l-lysine (PLL), observing not only a prominent effect on the rectification properties but also a higher sensitivity to external pH change than with noncoated nanopipettes. A similar strategy was also explored by Liu *et al.* using polyethyleneimines as building blocks to control the pH-dependent rectification properties of glass nanopipettes.^76^ In a similar vein, White and co-workers^77^ showed that the electrostatic fields at the orifice of a glass nanopore electrode modified with silane monolayers bearing amino groups could be manipulated by adjusting the solution pH. According to these authors, nanopore electrodes displaying small pore orifice radii exhibit a marked anion permselectively at pH < 4, whereas ion selectivity disappears at pH > 6. In 2007, Siwy and coworkers introduced a very interesting concept to control the pH-dependent rectification properties of conical solid state nanopore groups by creating positively and negatively charged domains on the nanopore walls.^78^ In this case the rectification properties exhibit a maximum value in the transition from acidic to alkaline pH values.

**Fig. 11:**
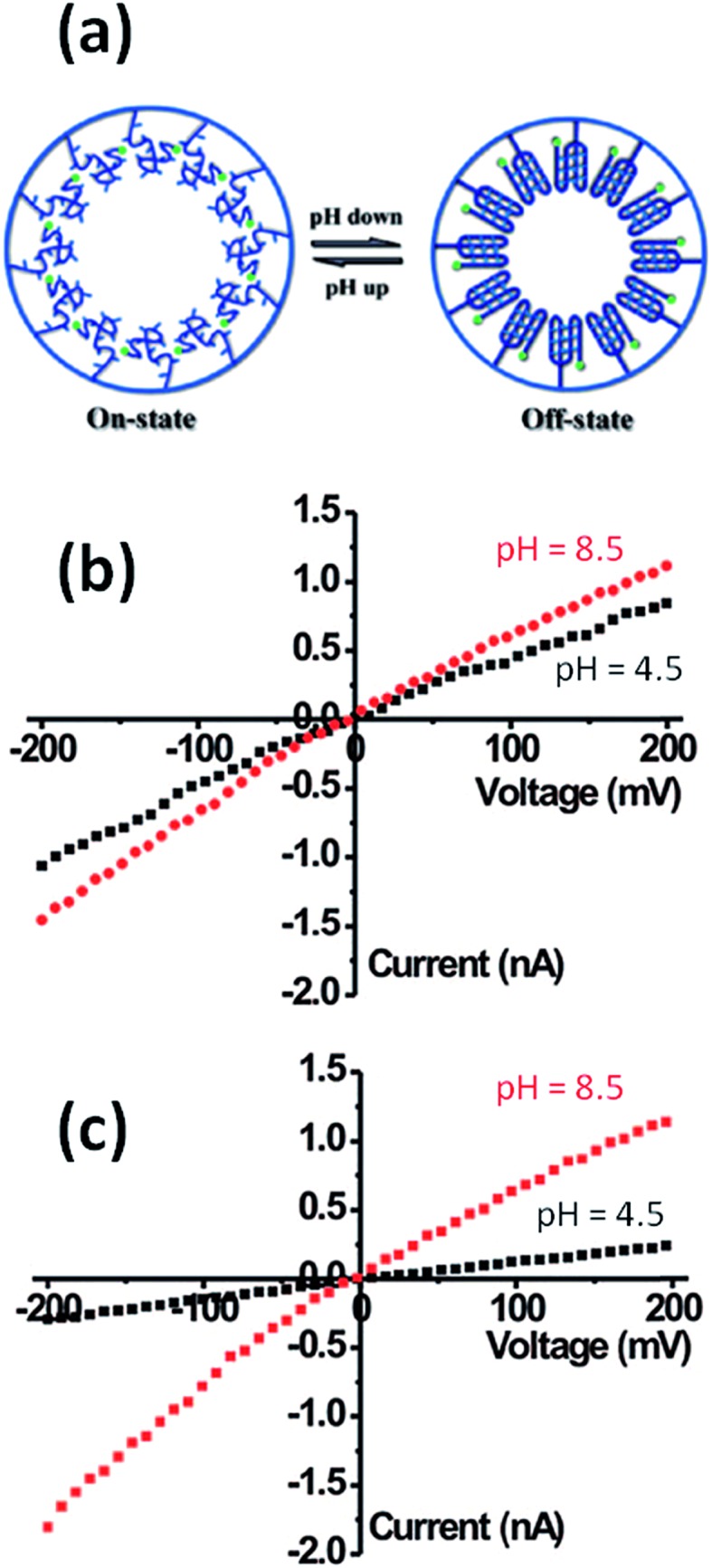
(a) Simplified cartoon displaying the conformational changes undergone by the DNA motor upon exposure to acidic conditions. *I*–*V* plots of an asymmetric single nanopore (b) before and (c) after attachment of motor DNA molecules onto the inner pore walls. Plots were recorded under different electrolyte conditions: (black) pH 4.5 and (red) 8.5. Reproduced with permission from Xia *et al.*, *J. Am. Chem. Soc.*, 2008, **130**, 8345–8350. Copyright 2008 American Chemical Society.

Later on, the scientific community started to explore the use of macromolecular and supramolecular entities as gating elements to adjust the pH-responsive properties of nanochannels. Jiang and workers demonstrated that the modification of single PET nanochannels with pH-sensitive DNA motors is a plausible route to create pH responsive nanofluidic devices.^79^ At low pH conditions, the DNA motors adopt a rigid quadruplex i-motif structure, thus decreasing the effective cross section of the nanochannel. However, at high pH values, the i-motif structure DNA motors unfold leading to a loosely single-stranded molecule bearing negative charges. This effect ultimately leads to an enhancement in the total ion conductance that, in turn, is transduced as an increase in the “ionic signal” (Fig. 11).

Polymer thin films provide a versatile toolbox to molecularly design interfaces with nanoscale control which are applicable to a plethora of “smart” chemical functionalities.^80–82^ If we consider that the degree of rectification is highly dependent on the surface charge density, the use of a responsive polymer layer can provide a simple means to sensitively increase the number of fixed charges on the nanopore walls.

**Fig. 12:**
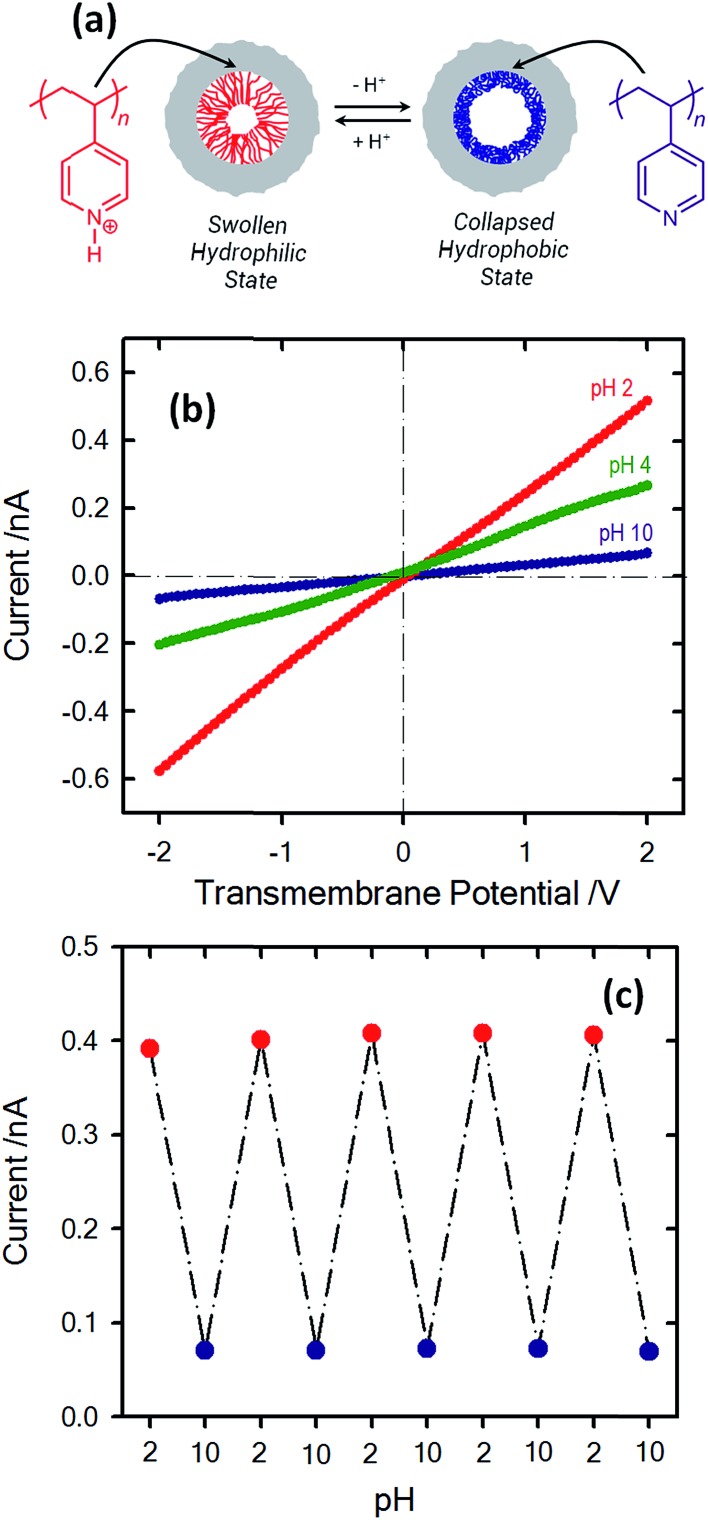
(a) Simplified scheme illustrating the conformational changes occurring in the PVP brush layer upon variation in the environmental pH. (b) *I*–*V* plots of a single cylindrical PVP brush-modified nanochannel in 0.1 M KCl at different pH values: (red circles) 2, (green circles) 4 and (blue circles) 10. (c) Reversible variation of the transmembrane ionic current passing through the PVP brush-modified nanochannel upon alternating the environmental pH. Reproduced with permission from Yameen *et al.*, *Nano Lett.*, 2009, **9**, 2788–2793. Copyright 2009 American Chemical Society.

**Fig. 13:**
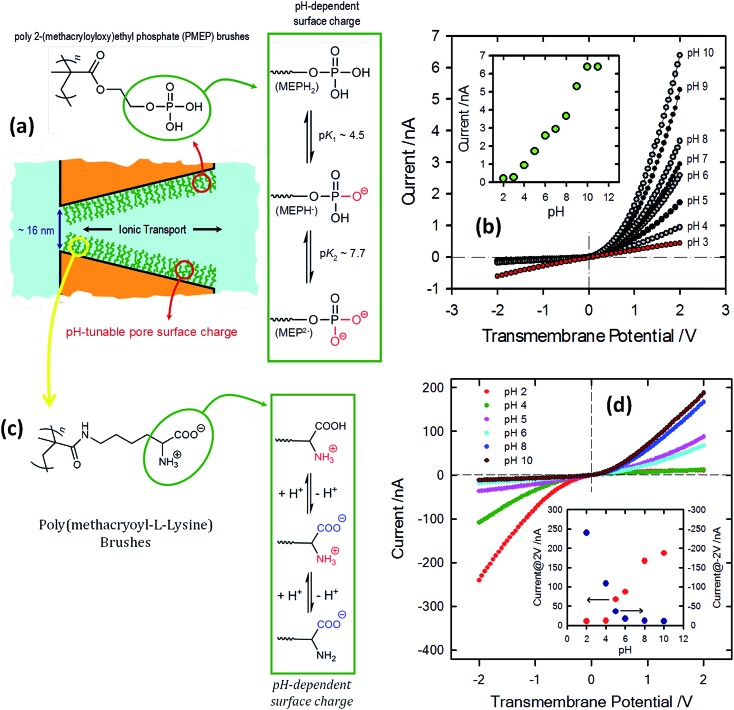
(a) Schematic depiction corresponding to the PMEP brush-modified conical nanochannel. The chemical structure of the PMEP polymer brush and the equilibrium associated with the pH-dependent behavior of the polyprotic monomer units are also indicated. (b) *I*–*V* curves corresponding to a single conical nanopore modified with PMEP brushes measured at different pH values (using 0.1 M KCl as an electrolyte). The inset describes the changes in the transmembrane currents measured at 2 V. Reproduced with permission from Yameen *et al.*, *Chem. Commun.*, 2010, **46**, 1908–1910. Copyright 2010 Royal Society of Chemistry. (c) Schematic diagram describing the PML brush-modified conical nanochannel. The chemical structure of the polymer brush and the equilibrium associated with the pH-dependent behavior of the zwitterionic monomer units are also indicated. (d) *I*–*V* curves corresponding to a single conical nanopore modified with poly(methacryloyl-l-lysine) brushes measured at different pH values (using 1 M KCl as electrolyte). The different pH values are displayed using colored symbols as indicated in the figure. The inset describes the changes in the rectified currents upon variation in the environmental pH. The red and blue dots refer to the rectified currents measured at 2 and –2 V, respectively. Reproduced with permission from Yameen *et al.*, *J. Am. Chem. Soc.*, 2009, **131**, 2070–2071. Copyright 2009 American Chemical Society.

Within this framework, Yameen *et al.* ^83^ developed proton-gated nanofluidic devices through the modification of cylindrical nanochannels with poly(4-vinylpyridine) brushes that ultimately act as gate keepers managing and constraining the flow of ionic species through the confined environment. The ionic current switching characteristics displayed by the nanochannels resembled the typical behavior observed in many biological channels that fulfill key pH-dependent transport functions in living organisms. Or, in other words, the nanochannels were switched from an “off” state to an “on” state in response to a pH drop (Fig. 12).^84^ This approach was also extended to the use of polyprotic brushes to create highly charged environments inside the asymmetric nanochannels with the aim of enhancing the rectifying behavior in the presence of subtle changes in proton concentration. Fig. 13 shows the *I*–*V* curves of conical PET nanochannels modified with poly(2-(methacryloyloxy)ethyl-phosphate) brushes under different pH conditions. It can be observed that the accurate control over the electrostatic characteristics of the nanochannel arising from the multiple protonation states of phosphate groups facilitates a strong effect on the rectification properties of the pore, thus reaching rectification efficiency values close to 50 (Fig. 13). The same group also demonstrated the construction of pH-tunable asymmetric single nanochannels with reversible rectification properties by grafting poly(methacryloyl-l-lysine) (PML) brushes on the inner surfaces of polyimide nanopores. The zwitterionic nature of the monomer units permits reversion of the surface charges by adjusting the solution pH above or below the isoelectric point of the methacryloyl-l-lysine. As a consequence, at low pH values the polymer brushes are positively charged and the nanopore displays the typical rectification properties of an anion selective nanodevice. However, at high pH values the inner walls become negatively charged and the nanochannels rectify the ionic transport in the opposite direction due to their cation-selective characteristics.^85^

The modification of single nanochannels with pH-responsive macromolecules has been extended by Jiang's group to the use of plasma-induced asymmetric modification of a symmetric hour-glass single nanochannel with polyacrylic acid (PAA) brushes in order to create pH-responsive nanodevices which can control, in a rather simple manner, both the asymmetric and gating ionic transport properties.^86^ This interesting behavior stems from the fact that PAA brushes located on one side of the asymmetric nanochannel promote a pH-modulated asymmetric distribution of surface charges across the nanochannel with a concomitant effect on the rectification properties. Along these lines, the asymmetric distribution of charged species on the pore walls as a result of asymmetric pH conditions has been proposed by Ali *et al.* as a strategy to modulate the rectifying functions of lysine-modified cigar-shaped nanochannels.^87^ More recently, Jiang and co-workers introduced a very interesting configuration to manipulate the transport properties of nanofluidic devices based on a cooperative pH response double-gate nanochannel.^88^ In this new configuration, polyvinylpyridine (PVP) and poly(acrylic acid) (PAA) layers were separately grafted onto the opposite ends (tips) of a cigar-shaped nanochannel *via* plasma-induced polymerization. When the pH is below a p*K*_a_ of 5.2, the PVP chains are in a swollen, hydrophilic cationic state. Conversely, above this p*K*_a_ the PVP chains are collapsed, neutral, and in a hydrophobic state. On the other hand, the PAA chains undergo a transition from a collapsed, neutral, hydrophobic state to a swollen, negatively charged, and hydrophilic state when the pH is increased above a p*K*_a_ of 4.7. In this context, both polymer gates can be actuated alternately/simultaneously under external symmetric–asymmetric pH stimuli. It is interesting to note that this double-gate configuration can mimic three key ionic transport features of biological ion pumps, including an alternating gate ion pumping process under symmetric pH stimuli, a transformation of the ion pump into an ion channel under asymmetric pH stimulation, and a fail-safe ion pumping feature under both symmetric and asymmetric pH conditions.

Due to the fact that most of the responsive molecules used for controlling the asymmetric transport of ionic species exhibit protonatable groups in their molecular structure, these systems can be ultimately regarded as *dual responsive* devices, with the proton concentration in solution acting as one of the stimuli. In many of these systems the second stimulus is an external physical input such as ultraviolet light,^89,90^ voltage^91,92^ or temperature.^93,94^ This feature has the interesting characteristic of allowing both control from the solution with the pH and from the outside or directly to the membrane with a complementary stimulus. It is important to note that these kinds of systems have been developed thanks to the combination of both physical and chemical modifications, to include advanced materials within nanofluidic structures. Some examples of these have already been described previously in this article, such as the use of conductive polymers or polymer brushes.^95^ In a similar vein, a combination of functional molecules can be used to achieve more complex responses by using two different stimuli;^96^ in this regard the ion pump developed by Zhang *et al.* is a clear example.^97^ An interesting illustration of dual responsive systems was provided by Li and co-workers employing conical nanopore channels modified with poly[2-(dimethylamino)ethyl methacrylate] (PDMAEMA) brushes. This macromolecular system undergoes both pH- and temperature-induced conformational transitions. As a result, a smart nanofluidic device that can be reversibly switched between the high conducting “ON” state and low conducting “OFF” state was obtained by varying the pH and temperature of the electrolyte. In particular, the PDMAEMA-modified nanopore system exhibited high gating efficiency upon switching between the “ON” state at pH 2, *T* = 50 °C and the “OFF” state at pH 12, *T* = 25 °C.

**Fig. 14:**
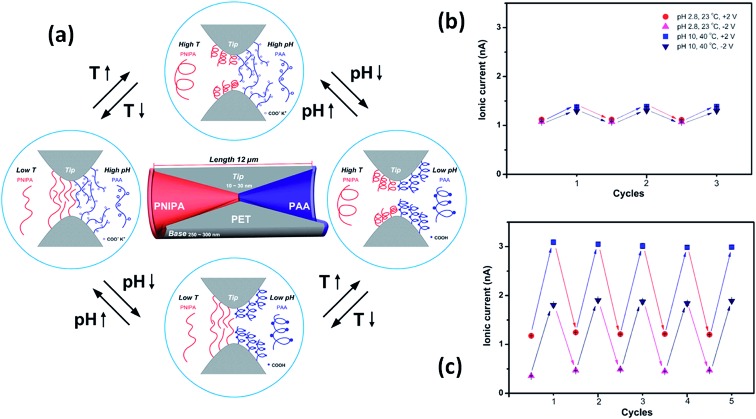
(a) Simplified cartoon describing the conformational and physicochemical changes taking place in the nanopore-confined PNIPAM and PAA layers upon varying the temperature and pH. Reversible switching of the ionic current transport of the single nanochannel prior to (b) and after (c) asymmetric chemical modification. Reproduced with permission from Hou *et al.*, *J. Am. Chem. Soc.*, 2010, **132**, 11736–11742. Copyright 2010 American Chemical Society.

Meanwhile, a more complex design presented by Hou *et al.* ^98^ includes the use of a symmetric double conical nanopore geometry modified asymmetrically with two different responsive polymers. One half of the pore was modified with poly(*N*-isopropylacrylamide) (PNIPAM) which is a temperature responsive polymer with a well-studied swelling transition at a temperature of 32 °C whilst the other half was modified with the pH sensitive polymer poly(acrylic acid) that has a p*K*_a_ of 4.7. This particular configuration allows fine tuning of the ionic current through the pores, both by changing the temperature and the pH of the electrolyte solution. Whilst PNIPAM produces gating of the current by swelling or deswelling at temperatures below and above the lower critical solution temperature, the PAA performs changes on the surface charge and therefore on the wettability of the pores as a function of the pH of the solution, thus generating rectified transport of ions. It was proven that both stimuli can be used synergically to enhance the response of the system (Fig. 14).

### Molecule-responsive nanochannels – chemical sensing and biosensing with switchable nanofluidic devices

The use of specific interactions to control the ionic transport properties of nanochannels is a key concept for developing sensing devices using nanofluidic elements. For example, it is possible to add chemical or biochemical selectivity by anchoring a predefined molecular receptor inside the nanopore.

Siwy *et al.* ^99^ reported one of the first attempts to develop protein nanobiosensors using asymmetric nanochannels. The sensing read-out consisted of passing an ionic current through the nanochannels. In order to achieve this goal, track-etched nanopores were firstly coated with gold by an electroless technique after which the nanopore was susceptible to modification by thiol gold chemistry. The molecular-recognition agents used for these modification procedures were biotin, protein-G and the antibody to the protein ricin. After modification, these pores became sensitive to streptavidin, immunoglobulin and ricin, respectively. Since the protein analyte and the channel mouth were comparable in size, binding of the protein led to blocking of the nanochannel, which was detected as a large reduction in the nanopore conductance.

Vlassiouk *et al.* ^100^ showed the construction of molecule sensitive devices using a bipolar diode (BP) approach that was presented in some previous work by the same group.^101^ BP implies the use of a strong asymmetric charge distribution inside the nanopores by differentially modifying the conical tip of the pore to increase the rectification and the output current. In this work, the tips of the pores were modified with biotin to respond to the presence of avidin and streptavidin. Furthermore, after the binding of proteins to the pore walls it was shown that their isoelectric point can be determined with current–voltage curves and that, with these, it is also possible to distinguish between both proteins. In order to explore the sensing capacities of this device, a similar procedure was employed by modifying the nanopore with the antibody of capsular poly-γ-d-glutamic acid (γ-DPGA) of *B. anthracis* instead. The resulting device was highly sensitive to the presence of γ-DPGA in solution.

**Fig. 15:**
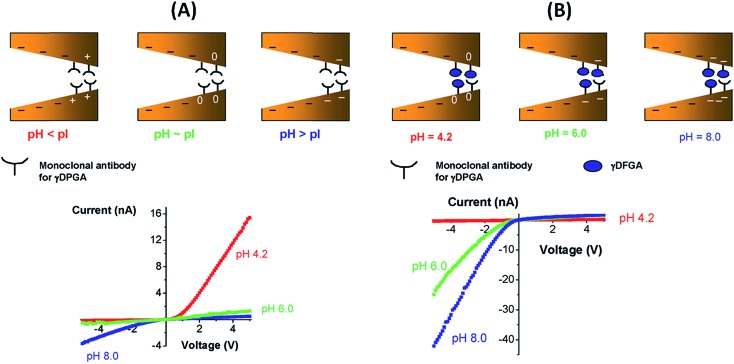
(A) (Top) Scheme describing the “iontronic” determination of the isoelectric point of the monoclonal antibody for the bacterial polyglutamic acid (γ-DPGA). (Bottom) Current–voltage plots corresponding to the monoclonal antibody-modified conical nanopore measured at different pH values. (B) (Top) “Iontronic” sensing of γ-DPGA using a monoclonal antibody-modified conical nanopore. (Bottom) Current–voltage plots corresponding to a γ-DPGA-conjugated conical nanopore. Depending on the pH conditions different surface charge densities are generated on the pore walls. Reproduced with permission from Vlassiouk *et al.*, *J. Am. Chem. Soc.*, 2009, **131**, 8211–8220. Copyright 2009 American Chemical Society.

Fig. 15 shows the experimental *I*–*V* plots corresponding to the development of the g-DPGA sensor. First, the tip of a single conical pore was modified with the monoclonal antibody (F26G3) to the bacterial γ-DPGA. The nanofluidic sensor bearing the antibody at its tip exhibited a strong dependence of its *I*–*V* behavior on the pH of the bulk solution (Fig. 15). Under alkaline conditions the rectification direction was similar to that of the bare pore, thus revealing that the antibody was negatively charged. Under acidic conditions the rectification was reversed and increased, thus indicating that the bipolar diode junction was formed and the antibody was positively charged. Meanwhie, antibody-modified pores incubated with bacterial γ-DPGA exhibited drastic changes in their rectification properties. Carboxyl groups of γ-DPGA confer a strong anionic character to this polypeptide for pH > 4, when these groups are ionized. As a result, the biorecognition process yields a system that rectifies the ionic current in one direction for a broad range of pH conditions.

**Fig. 16:**
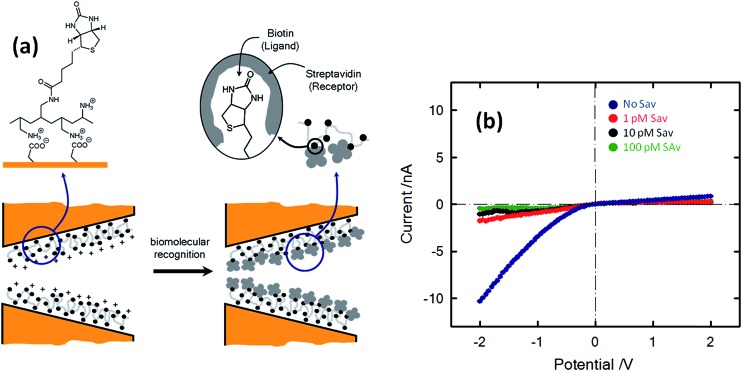
(a) Simplified depiction of the dimensional changes occurring in the nanopore tip during and after the biorecognition process. (b) Current–voltage plots of a biotin-modified single conical nanopore in 0.1 KCl in the presence of different concentrations of streptavidin (SAv): (blue) no SAv; (red) 1 pM; (black) 10 pM; (green) 100 pM. Reproduced with permission from Ali *et al.*, *J. Am. Chem. Soc.*, 2008, **130**, 16351–16357. Copyright 2008 American Chemical Society.

The concept of biosensing with nanopores was further extended by Azzaroni and co-workers^102^ through the incorporation of biorecognition elements into conical nanochannels using electrostatic self-assembly. This strategy is based on the use of bifunctional macromolecular ligands to electrostatically assemble biorecognition sites into the nanochannel wall, which can then be used as recognition elements for constructing a nanobiosensor. In this case the bifunctional macromolecular ligand was polyallylamine labeled with biotin moieties (b-PAH). Fig. 16 depicts the *I*–*V* plots corresponding to biotin-modified nanopores in contact with different streptavidin solutions. It is observed that the presence of streptavidin, even at very low concentrations, promotes a marked change in the rectified ionic current. In the presence of 1 pM streptavidin the rectified ionic current decreased from –10.3 nA to –1.7 nA. This change reveals that the blockage of the nanopore due to the formation of the bioconjugate decreased the ionic flux across the nanopore by ∼85%. This effect is even more pronounced in the presence of more concentrated SAv solutions. For example, the presence of 100 pM SAv prompted a ∼96% decrease in the rectified current observed in the nonbioconjugated nanopore. Experimental results demonstrated that ligand-modified nanochannels functionalized *via* electrostatic assembly are very stable and the biorecognition event does not promote the removal of the ligands from the channel surface. In addition, control experiments indicated that the electrostatically assembled nanochannel surface displays good biospecificity and nonfouling properties.

Ali *et al.* also explored the supramolecular bioconjugation of the concanavalin A (Con A) protein with the glycoenzyme horseradish peroxidase (HRP) inside a single cylindrical nanochannel *via* lectin-carbohydrate recognition processes.^103^ The bioconjugation of Con A inside the nanochannel brought about a reduction in the effective area for ionic transport, and consequently this blocking effect was exploited to tune the conductance and selectivity of the nanochannel in aqueous solution.

In a pioneer study, Heins *et al.* explored sensing of porphyrin molecules using conical polyimide nanopores. The diameter of the small opening of the nanopore was comparable to the size of the molecule, therefore every time a molecule passed through the pore driven by the voltage difference across the membrane, an increase in the resistance of the pore was produced. The increase in the pore resistance was observed as a sharp decrease in the ionic current.^104^ Using polyethyleneterephthalate (PET) conical nanopores, Guo *et al.* described a series of experiments for evaluating the sensing capacity of this type of nanopore with molecules of different sizes and hydrophobicities. In these experiments, Hoechst 33342, propidiumiodide and bupivacaine were used as analytes. The outcome of these assays was that ion-track etched polymer nanopores are better suited for sensing smaller and more hydrophobic molecules. In this case the highest binding constant, which correlates with a lower detection limit, was found for Hoechst 33342. An interesting aspect of this report is that experiments were not performed using the typical stochastic sensing procedure of resistive pulse measurements but using current–voltage characteristics. Since the analytes used were cationic, they bind strongly to the surface of the pores changing the surface charge from negative to positive, reverting the rectification of the ionic current.^105^

A common problem with the use of non-modified nanopores is the lack of selectivity towards specific small molecules. Different research groups have examined the use of covalent and noncovalent chemistries increase their selectivity. In the case of noncovalent chemistry there is an interesting report from Wen *et al.* that shows the fabrication of an acetylcholine sensor using a PET nanopore modified through a layer-by-layer (LbL) procedure in which successive layers of polyethyleneimine and sulfonatocalixarene (SCX) were assembled on the pore surface. Due to the fact that the calixarene molecules interact strongly with cationic organic molecules, the LbL modified nanopore responds to the presence of such molecules such as, for example, acetylcholine (Ach). Since Ach bears positive charges, the inclusion of this molecule in the nanopore increases the positive charge surface density, increasing the rectification of the ionic current as well.^106^ The same group also extended the use of nanopores modified with calixarenes to the detection of arginine.^107^

**Fig. 17:**
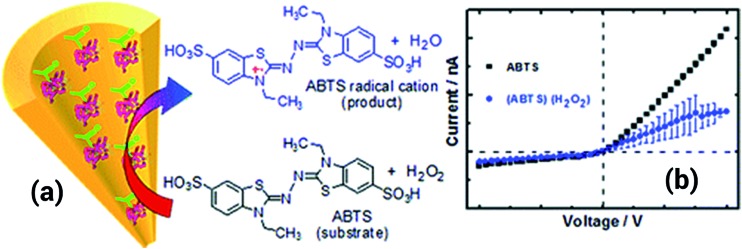
(a) Cartoon showing the formation of the ABTS radical cation upon the reaction with hydrogen peroxide in solution. (b) *I*–*V* curves measured for a horseradish peroxidase modified nanopore with and without hydrogen peroxide in solution, the substrate ABTS was always present in solution. Reproduced with permission from Ali *et al.*, *Anal. Chem.*, 2011, **83**, 1673–1680. Copyright 2011 American Chemical Society.

Another approach including the use of polyethyleneimine (PEI) was proposed by Ali *et al.* ^108^ In this case, the PEI was covalently attached to the nanopore by a carbodiimide coupling process. Once the PEI was attached to the pore walls, the resulting nanopore was used for recognition of adenosintriphosphate (ATP) due to the strong interactions present between primary amines and phosphates. When ATP was introduced into the solution, the conductance of the pore decreased significantly due to a partial blocking of the pore as well as a neutralization of the positive charges that originated from the PEI layer. The lack of reversibility represents an important drawback of this system; once the ATP is fixed is not possible to restore the system to its initial state. Using a rather similar coupling procedure, the same group managed to decorate the nanopore surface with horseradish peroxidase (HRP) to build a hydrogen peroxide sensor capable of detecting concentrations down to 10 nM. In order to sense hydrogen peroxide a substrate, such as 2,2′-azino-bis(3-ethylbenzothiazoline-6-sulfonate) (ABTS), was required to be included into the solution. When the peroxide is present in the solution the HRP attached to the pore surface decomposes the molecules forming radicals that are eventually transferred to the ABTS. In this way the enzymatic process generates cationic radicals that ultimately change the charge of the nanopore with a concomitant effect on the ionic transport properties. Due to the fact that the system is responsive to the concentration of both species, *i.e.* ABTS and H_2_O_2_, is necessary to fix the concentration of ABTS in order to sense the hydrogen peroxide (Fig. 17).^109^

Rant and co-workers^110^ show that metallized silicon nitride nanopores chemically modified with nitrilotriacetic acid (NTA) receptors can be used for the stochastic sensing of proteins. NTA molecules interact with Ni ions that further interact with His-tagged proteins in order to grant the nanopore a particular biochemical functionality. By this method, the pore can interact with other molecules by specific interactions. To demonstrate the versatility of their approach, the authors proved the detection of His-tagged proteins as well as discriminating between the subclasses of rodent IgG antibodies. In a rather similar way, Tahir *et al.* used the specific interactions of NTA with His-tagged silicatein to tune the rectification and conductance of the pores. Through this approach, the authors showed that the isoelectric point of the molecule can be obtained from current–voltage measurements.^111^ Sun *et al.* ^112^ reported the fabrication of cysteine-responsive biomimetic single conical PET nanochannels by a thiol–yne reaction strategy. The approach involved the formation of strong covalent bonds on propargylamine-functionalized nanochannels through a photo-initiated thiol–yne click reaction. Thus, even in complex matrices, this system shows a highly specific response and no interference. To this end, a photoelectrochemical cell was constructed so that the sample could be irradiated from both sides. After modification with propargylamine, the channel surface is covered by –yne and some remaining –COO^–^ groups. At acidic pH values the nanochannel surface bears no effective charges, so the ionic current is rather low. On the other hand, when the thiol–yne reaction takes place, the surface becomes positively charged under acidic conditions, hence this process leads to a reversal of the rectifying characteristics.

**Fig. 18:**
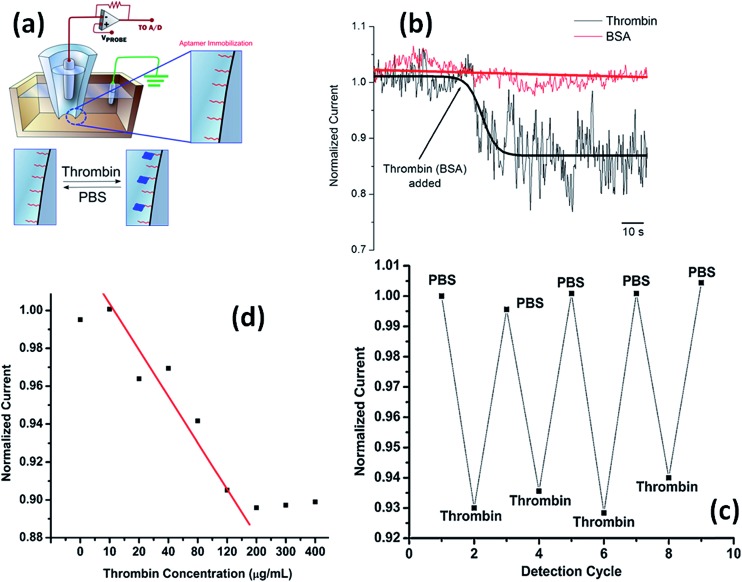
(a) Schematic representation of the experimental setup employed for sensing thrombin using an aptamer-functionalized quartz nanopipette. (b) Variation of the normalized ionic current (“iontronic” signal) upon thrombin (150 mg mL^–1^) and BSA (150 mg mL^–1^) interaction with the aptamer-functionalized quartz nanopipette in phosphate-buffered saline solution (pH 7.4). (c) Regeneration of the nanopipette sensor after binding of thrombin. (d) Response of the aptamer-functionalized nanopipette sensor to increasing concentrations of thrombin. Reproduced with permission from Actis *et al.*, *Biosens. Bioelectron.*, 2011, **26**, 4503–4507. Copyright 2011 Elsevier B.V.

Actis *et al.* ^113^ have demonstrated the selective thrombin detection using aptamer-functionalized quartz nanopipettes (Fig. 18). The interaction of thrombin with its specific aptamer tethered on the nanopipette causes a partial occlusion of the pore thus promoting a detectable decrease in the ionic current. Control experiments using BSA revealed no changes in the normalized current, indicative of the selectivity of thrombin binding in the nanopore. The bound thrombin can be removed by washing the nanopore with buffer. These authors also showed a direct correlation between the ionic signal change and the analyte concentration in the bulk solution in the range 10–200 mg mL^–1^. The same group also explored this approach for the ultrasensitive detection of mycotoxins using nanopipettes modified with the respective antibodies. Experimental results showed that nanopipette sensors are capable of detecting mycotoxins with a detection limit as low as 100 fg mL^–1^ and a linear range of 3 logs.^114^

**Fig. 19:**
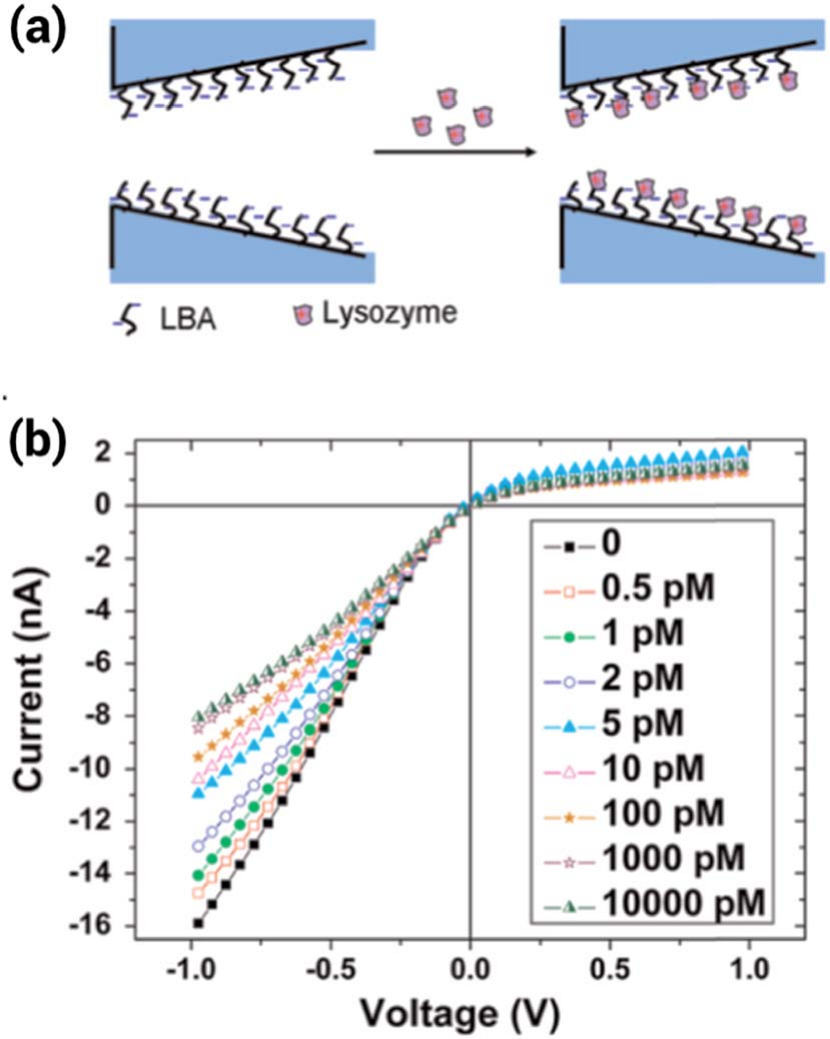
(a) Scheme showing the interaction of the LBA-modified nanopore with the lysozyme and (b) *I*–*V* curves for an LBA-modified nanopore with different concentrations of lysosyme from 0 to 10 000 pM. Reproduced with permission from S.-L. Cai *et al.*, *Biosens. Bioelectron.*, 2015, **71**, 37–43.

Another example of sensing proteins using aptamer–protein interactions has been recently shown by Cai *et al.* ^115^ In this work, the authors covalently bound a lysosyme binding aptamer (LBA) to a glass conical nanopore through silane chemistry, and found a remarkable sensitivity to lysosyme using concentrations as low as 0.5 pM. The binding of the lysozyme reduced the effective charge of the surface therefore decreasing the rectification efficiency of the nanopore (Fig. 19). The specificity of the aptamer–lysosyme interaction was proven using several different proteins, such as BSA, pepsin and cytochrome C.

In addition to nucleic acids and proteins, carbohydrates play critical roles in multiple biological functions and physiological processes, thus, the development of strategies to detect these molecules has become increasingly important. Different groups have reported the use of single nanochannels decorated with boronic acid receptors with the aim of detecting saccharides.^116,117^ Boronic acids exhibit the ability to reversibly bind carbohydrates resulting in conversion of the boronic acid to an anionic boronate ester when the p*K*_a_ is shifted to a value lower than the pH of the environment. In most cases, the saccharide response in boronic acid-modified nanopores resulted in moderate changes in current rectification. This issue has been addressed in detail by Vilozny *et al.* ^118^ through the integration of polymeric building blocks that can respond to saccharides not only by changing charge but also their conformation. These authors employed poly(vinylpyridine) quaternized with benzylboronic acid groups to modify the nanochannel walls; this is an interesting cationic polyelectrolyte that undergoes a transition from a swollen to a collapsed state upon binding to monosaccharides. As a result, the current rectification in nanopipettes can be reversibly switched depending on the concentration of monosaccharides. Such molecular actuation of nanofluidic conductance may be used to engineer feedback-controlled delivery systems provided that the selectivity can be tuned for different carbohydrates.

Chiral recognition of l-tryptophan (l-Trp) using beta-cyclodextrin-modified biomimetic single nanochannels has been recently reported by Xie *et al.* ^119^ The operational principle of this nanodevice relies on the binding of Trp by embedding a hydrophobic aromatic ring into the hydrophobic cavity of the cyclodextrin and exposing the polar part outwards to interact with the hydroxyl groups and form hydrogen bonds. The difference in affinity between both enantiomers results in selective binding of the l-enantiomer, thus granting the ability of chiral recognition to the nanochannels. Chiral discrimination was demonstrated by measuring the transmembrane ionic current of β-CD-modified nanochannels in the presence of each enantiomer. In the presence of solutions containing l-Trp the binding of the amino acid to cyclodextrin changed the surface wettability, producing changes in the transmembrane ion current. Conversely, a similar experiment performed in the presence of d-Trp or the other aromatic amino acids revealed no changes in the ionic current. More recently, Li's group reported the chiral recognition of arginine on label-free polymeric nanochannels by adding bovine serum albumin (BSA) as a chiral selector.^120^ These results based on the use of chiral receptors and selectors open up a promising path towards developing nanofluidic devices for applications in drug detection and analysis.

Of all the applications of nanofluidic systems, biosensing is perhaps one of the most actively pursued.^11,121–123^ The proven ability of nanopores to sense single molecules, to differentiate between different molecules and even to show chemical changes in the same type of molecule is the reason why nanofluidic devices are taking center stage in biosensing, especially in the area of DNA sensing and sequencing.^124^ There are already commercially available devices for sequencing DNA which are fully based on biological nanopores, that have already reached important milestones such as sequencing bacterial DNA from *E. coli* and viral DNA from the Ebola virus.^125,126^ The latter of which had the interesting characteristic of the experiments being carried out *in situ*, showing the portability and speed of this new generation of sequencing device. However, there is wide interest in developing fully abiotic systems in order to avoid handling and manipulation of protein channels.

## 4. Summary and outlook – Mother Nature keeps leading us down new paths

“*Naturae enim non imperatur nisi parendo*” (“*Nature can only be commanded by obeying her*”) Francis Bacon, *Novum Organum I*, 129.

Much of the inspiration to design, construct and operate nanofluidic devices stems from the outstanding progress of molecular biology and biophysics that has begun to unveil the secrets of channel proteins which constitute essential biological machineries. In general, channel proteins act as “control stations”, regulating transport through pores, thus operating as a gate. The intricacy of biological systems cannot be overestimated; as a consequence, the bottom-up construction of devices as complex as those present in nature turns into a prohibitive task. Over the years, the creativity of chemists, physicists and materials scientists provided a broad variety of means for developing “abiotic” nanoarchitectures displaying ion transport and gating properties similar to those observed in biological pores. If we consider the early developments related to the Coulter counter and patch-clamp technique, the advent of these biomimetic nanofluidic devices heralded a new beginning for “iontronics”. It is particularly encouraging that this scientific field is now endorsed by many research groups around the world, since technological progress has always been related to the ingenuity of scientists and engineers.

A careful look at the literature reveals that, unexpectedly, this field of research, soundly supported by rigorous physical backgrounds, glided furtively from pure curiosity towards much more concrete and tangible domains. We think that a large part of the merit of this evolution is that scientists overcame the fences in which they were traditionally confined and started to collaborate across other disciplines – chemistry going hand in hand with physics and biology. It is delightful to see that cross-discipline exchange in nanopore research is now possible like it has never been before.

In this perspective article, we have discussed different examples in which solid-state nanopores equipped with molecular-scale components endowed with sensing and switching functions can change their ion transport characteristics in response to external stimuli. Electrical fields, temperature changes, pressure, light, pH changes, or biochemical signals can be used as external effectors. Upon stimulation, these responsive units undergo particular rearrangements of a chemical or physical nature, which ultimately govern the interaction between the passing ions and the pore walls.

Molecular components suitable for this task might exhibit two or more physico-chemically distinct states that can be conveniently addressed or switched by application of external triggers to transduce a particular signal. We should note that the concept of “signaling”, in the case of nanopores, refers to physico-chemical processes that are generated in response to a particular stimulus and that can be detected by measuring the ionic current passing through the pore. Regarding the latter, we should bear in mind that confinement inside a nanopore can significantly change the interaction between molecules and their surroundings.^127^ In confined spaces of nanoscopic dimensions, all the molecular counterparts are in close interaction with the pore surface, leading to remarkable – and exciting – consequences in their physical and chemical properties.^128^ It is well known that molecular systems confined in nanoscale geometries show structures, dynamics and even chemical properties^129^ different from those exhibited in bulk. As such, the richness of chemical phenomena in confined environments could be further exploited to introduce advanced functionalities in nanofluidic elements.

The precise incorporation of active elements into nanochannels and nanopores is the cornerstone of the rational design of “on-demand” nanofluidic devices for multiple applications. Over the past several years, we have witnessed the appearance of different strategies enabling the formation of organic, polymeric, supramolecular and biological assemblies inside nanopores, and this trend is still continuing. At present, more and more functional units become eligible for incorporation into the pore walls, thus adding new capabilities to the nanofluidic architectures. An essential aspect of the molecular design and operation of these devices is that the incorporated molecular functions need to exert a controllable strong influence over the local interactions that take place in the ionic environment of the nanochannel. The numerous applications of these ionic devices strongly depend on several factors concerning this matter. This is particularly relevant if we consider that application potential of responsive solid-state nanopores is mostly influenced by: (i) the degree of change in the nanopore surface properties after the external trigger is applied, (ii) the rate at which this change or switch occurs, (iii) the reversibility of the switching process, (iv) the possibility to repeat the switching operation in cycles, (v) the time scale needed to complete a switching cycle, (vi) the relationship between the magnitude of the applied stimuli and the magnitude of the generated ionic signal, and (vii) the ratio between ionic signals in “ON” and “OFF” states, *i.e.* gating efficiency. An ideal responsive nanopore would be one that exhibits instantaneous and drastic variation in ionic currents upon application of the external stimulus. Depending on the application, it is also necessary to modulate the ionic response with the strength of the applied field (“tunable” nanopores), to control the reversibility of the imposed changes (“switchable” nanopores), or to display independent response to different stimuli (“multifunctional” nanopores).

For these purposes, two main approaches have been employed: (i) the integration of recognizable supramolecular or biological units and (ii) the integration of functional groups that fine-tune the pore's hydrophilicity and/or surface charge. The first approach is the core concept behind the use of nanofluidic elements as sensing devices. The second one is the cornerstone on which gateable nanofluidic devices are built. In both cases a facile handling of signal read-out with readily available electrical/electrochemical equipment can reveal, through variations in the *I*–*V* response, the presence of a particular physical, chemical or biological signal.

We believe that the next step in the design and operation of nanofluidic devices is their modification with multicomponent molecular systems capable of performing concerted (signaling) functions with different levels of sophistication. Our hypothesis relies on the fact that new dimensions and capabilities can arise from synergisms between specifically designed molecular building blocks and the implementation of advanced supramolecular notions in confined environments. This is particularly enthralling as chemists have no hesitation in putting whatever they believe could be useful in nanopores to achieve their goals. However, there is still a long way to go to fully understand multifunctional supramolecular interactions in nanopore surfaces and to utilize them in a rational manner. In this context, it is important to highlight that novel conceptual interpretations of old classes of chemical reactions can also open new scenarios regarding the potential of integrated chemical systems for signal-processing in nanofluidic elements.

The new horizons for sensing and signal transduction provided by nanopore systems appear very wide and the future offers the prospect of many developments as materials scientists show an increased mastery in the design and construction of inorganic and polymeric nanochannels of different dimensional characteristics. Yet, there is a need to keep exploring new avenues to attain nanofluidic elements exhibiting enhanced gating functions. This is the cornerstone to converting molecular functions, built-in within the nanopore, into addressable nanofluidic devices displaying measurable ionic signals in the presence of specific stimuli.

Looking back at what has been done over these past few years, it is easy to see that the field has reached a new level of maturity. And yet, with the vast repertoire of synthetic strategies at our disposal to create new nanopore structures with new, and perhaps unpredictable properties, we can expect exciting discoveries to continue in this dynamic field. With this in mind, we hope that this review can trigger a cascade of refreshing ideas in nanopore-based sensing devices as well as assist in the rational design of gateable nanofluidic elements.
